# In Vitro Inhibition of Digestive Enzymes by Leaf Metabolites from *Neurolaena lobata* (L.) R. Br. ex Cass. (Asteraceae)

**DOI:** 10.3390/molecules31152596

**Published:** 2026-07-25

**Authors:** Yohum Lozada-Diaz, Oscar J. Patiño-Ladino, Juliet A. Prieto-Rodríguez

**Affiliations:** 1 Departamento de Química, Facultad de Ciencias, Pontificia Universidad Javeriana, Bogotá 110231, Colombia; y-lozada01@javeriana.edu.co; 2 Departamento de Química, Facultad de Ciencias, Universidad Nacional de Colombia, Sede Bogotá, Bogotá 111321, Colombia; ojpatinol@unal.edu.co

**Keywords:** *Neurolaena lobata*, digestive enzyme inhibition, pancreatic lipase, α-glucosidase, sesquiterpene lactones, methylated flavonols

## Abstract

Digestive enzymes such as pancreatic lipase (PL), α-glucosidase (AG), and α-amylase (AA) play key roles in the hydrolysis of dietary lipids and carbohydrates and are therefore relevant biochemical targets for evaluating compounds with potential to modulate postprandial metabolic responses. *Neurolaena lobata* (L.) R. Br. ex Cass. (Asteraceae) is a neotropical medicinal species traditionally used for several health-related conditions, including metabolic complaints. This study evaluated the in vitro inhibitory activity of the hydroalcoholic extract, selected VLC fractions, and isolated metabolites from *N. lobata* leaves against PL, AG, and AA. Enzyme inhibition screening of the extract and VLC fractions was used to select fractions for phytochemical investigation, affording six known metabolites: the sesquiterpene lactones neurolenin B (**C1**) and lobatin A (**C2**), the benzoic acid derivatives p-hydroxybenzoic acid (**C3**) and 3,4-dihydroxybenzoic acid (**C4**), and the flavonoids 6-hydroxykaempferol 3,7-dimethyl ether (**C5**) and quercetagetin 3,7-dimethyl ether (**C6**). The hydroalcoholic extract and the MeOAc and iPrOH fractions inhibited PL, AG, and AA. Among the isolated compounds, **C1**, **C2**, **C5** and **C6** inhibited PL and AG, with IC_50_ values ranging from 134 to 615 µM and from 170 to 639 µM, respectively, whereas all compounds showed weak AA inhibition. Exploratory kinetic analysis yielded apparent inhibition profiles mainly consistent with competitive behavior; **C6** showed an apparent profile consistent with noncompetitive inhibition against AG under the assay conditions used. These findings expand the phytochemical and in vitro bioactivity profile of *N. lobata* and provide preliminary evidence of digestive enzyme modulation in isolated enzyme systems, without establishing therapeutic efficacy.

## 1. Introduction

Metabolic syndrome comprises a cluster of interrelated metabolic alterations, including central obesity, impaired glucose homeostasis, hypertension, and atherogenic dyslipidemia, which collectively increase the risk of type 2 diabetes mellitus (T2DM) and cardiovascular disease [1,2]. Among these alterations, postprandial hyperglycemia and postprandial hyperlipidemia are particularly relevant because they reflect the rapid intestinal absorption of dietary carbohydrates and lipids and contribute to metabolic stress, especially in individuals with insulin resistance, obesity, or T2DM. Exaggerated postprandial glucose and triglyceride responses may further promote oxidative stress, low-grade inflammation, endothelial dysfunction, and worsening insulin sensitivity [3,4]. Because the intestinal availability of absorbable monosaccharides and fatty acids depends largely on the hydrolysis of dietary carbohydrates and triacylglycerols, digestive enzymes involved in these processes represent relevant biochemical targets for modulating nutrient absorption [5,6,7]. Pancreatic lipase (PL) catalyzes the hydrolysis of dietary triacylglycerols into absorbable fatty acids and monoacylglycerols, whereas α-amylase (AA) hydrolyzes starch into smaller oligosaccharides, and α-glucosidase (AG) participates in the final release of absorbable monosaccharides [6,8,9].

Several oral drugs used in the management of T2DM and obesity act by inhibiting digestive enzymes involved in carbohydrate and lipid absorption. Acarbose and miglitol inhibit intestinal α-glucosidases, delaying carbohydrate digestion and reducing postprandial glucose excursions, whereas orlistat inhibits gastric and pancreatic lipases, thereby decreasing the hydrolysis and intestinal absorption of dietary lipids [8,10]. Although these drugs validate digestive enzyme inhibition as a clinically relevant strategy, their use may be limited by moderate efficacy, gastrointestinal adverse effects, tolerability issues, and the multifactorial nature of obesity, T2DM, and metabolic syndrome [8,10,11]. These limitations have encouraged the biochemical screening of natural products as potential modulators of digestive enzymes. In this context, medicinal plants are of particular interest because their secondary metabolites, including phenolic compounds, flavonoids, terpenoids, alkaloids, and other structurally diverse phytochemicals, may exhibit digestive enzyme-modulating activity [11,12,13,14]. Traditional medical knowledge can therefore serve as a rational starting point for selecting plant species for phytochemical and biological studies, in line with the WHO Global Traditional Medicine Strategy 2025–2034, which emphasizes evidence generation, safety, quality, appropriate use, and respect for cultural diversity and biodiversity [15]. Accordingly, the evaluation of PL, AA, and AG inhibition in plant extracts, fractions, and isolated metabolites represents a useful in vitro strategy to identify plant-derived compounds capable of modulating lipid and carbohydrate digestion.

*Neurolaena lobata* (L.) R. Br. ex Cass. is a medicinal species of the family Asteraceae with a neotropical distribution, occurring in Central and South America and the West Indies, where it typically grows as a subshrub or shrub in seasonally dry tropical habitats [16]. This species has been traditionally used in Central America, the Caribbean, and northern South America for different health-related purposes, including inflammatory conditions, gastrointestinal disorders, parasitic infections, wound healing and metabolic complaints [17,18,19]. Previous pharmacological studies have reported hypoglycemic activity for ethanolic extracts of *N. lobata* leaves in murine models, supporting the scientific interest of this species in studies related to glucose metabolism [17,18]. Nevertheless, the secondary metabolites that may contribute to the biological effects attributed to this plant remain insufficiently characterized, particularly in relation to digestive enzyme modulation.

From a phytochemical perspective, *N. lobata* is mainly characterized by the presence of sesquiterpene lactones, including germacranolides, seco-germacranolides, furanoheliangolides, and related terpenoids [19,20,21,22]. These metabolites are especially relevant within Asteraceae and have been associated with several biological properties, including anti-inflammatory, antiproliferative, and antiprotozoal activities [19,20,21]. In addition to sesquiterpene lactones, flavonoids have also been reported in *N. lobata* and related *Neurolaena* species, including 6-hydroxy- and 6-methoxyflavonoids, which may contribute to the chemotaxonomy and bioactivity profile of the genus [23,24]. Despite these advances, most studies on *N. lobata* have focused on the isolation of metabolites and the evaluation of selected biological activities, while the relationship between its phytochemical composition and the inhibition of digestive enzymes involved in lipid and carbohydrate hydrolysis remains poorly explored.

Therefore, this study aimed to expand the phytochemical and in vitro bioactivity profile of *N. lobata* by characterizing metabolites from selected enzyme-inhibiting leaf fractions and evaluating their inhibitory activity against PL, AA, and AG in isolated enzyme systems.

## 2. Results and Discussion

### 2.1. Enzyme Inhibition of the HA Extract and VLC Fractions Against PL, AA, and AG

The hydroalcoholic extract (HA) and VLC fractions obtained from *N. lobata* leaves showed differential inhibitory effects against pancreatic lipase (PL), α-amylase (AA), and α-glucosidase (AG) (Table 1). These data indicate that PL and AG inhibition in *N. lobata* leaves was concentrated mainly in the HA extract and in the intermediate-polarity MeOAc and iPrOH fractions, whereas the stronger activity of HA than of the individual fractions may reflect compositional differences between the extract and the individual fractions. Compared with the previously cited AG inhibitory effect of a *N. lobata* hydroalcoholic extract IC_50_ value of 886 µg/mL [17,18], the present HA extract showed a lower IC_50_ value under the assay conditions used. Such a difference is plausible because apparent AG potency is sensitive to both extract composition and assay design, including plant provenance and harvest conditions, extraction procedure, enzyme source, substrate, incubation time and solvent composition. To the best of our knowledge, this is the first report of the inhibitory activity of the HA extract and its VLC fractions against AA and PL, and of the VLC fractions against AG. Based on their inhibitory profile, the MeOAc and iPrOH fractions were selected for phytochemical investigation (Figure S1).

### 2.2. Phytochemical Study

**Figure 1:**
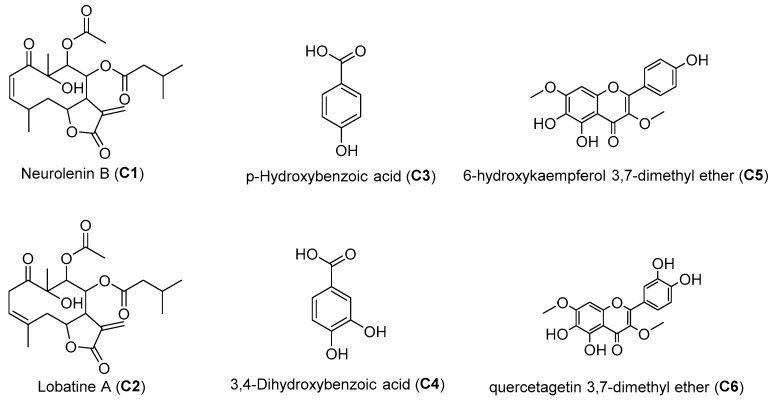
Chemical structures of compounds isolated from selected active fractions of *N. lobata* leaves.

Phytochemical investigation of the selected MeOAc and iPrOH fractions from *N. lobata* leaves afforded six known metabolites (Figure 1), which were grouped into three main structural classes: the germacranolide-type sesquiterpene lactones neurolenin B (**C1**) and lobatin A (**C2**); the benzoic acid derivatives p-hydroxybenzoic acid (**C3**) and 3,4-dihydroxybenzoic acid, also known as protocatechuic acid (**C4**); and the methylated flavonols 6-hydroxykaempferol 3,7-dimethyl ether (**C5**) and quercetagetin 3,7-dimethyl ether (**C6**). The structures were assigned by 1D and 2D NMR spectroscopy and by comparison of the obtained spectroscopic data with published values (Figures S2–S19). The isolated metabolite profile is consistent with previous phytochemical studies reporting *N. lobata* as a rich source of sesquiterpene lactones, particularly germacranolide and furanoheliangolid-type compounds.

The sesquiterpene lactones **C1** and **C2** have been previously isolated from *N. lobata* and are consistent with the well-documented sesquiterpene lactone-rich profile of this species. Their NMR data showed diagnostic features of germacranolide-type sesquiterpene lactones, including lactone carbonyl signals, resonances associated with the α-methylene-γ-lactone moiety, oxygenated methine carbons, ester carbonyls, and characteristic olefinic signals. The close structural relationship between **C1** and **C2** was reflected in their NMR profiles, while selected diagnostic signals allowed their differentiation. Neurolenin B (**C1**) was characterized by two olefinic proton signals at δH 6.58 and 6.00, associated with the conjugated enone system, whereas lobatin A (**C2**) showed methylene signals for H-2, an olefinic H-3 resonance, and a quaternary olefinic carbon assigned to C-4, indicating a different arrangement of the unsaturated system. Additional differences were observed in the oxygenated/acylated region, with **C1** showing closely overlapping H-8/H-9 resonances, while **C2** displayed more clearly differentiated H-8 and H-9 signals. The main COSY, HSQC, and HMBC correlations supporting these assignments are summarized in Figures S4–S6 and S9–S11, and the corresponding 2D NMR spectra are provided in the Supplementary Materials.

Previous phytochemical studies have shown that *N. lobata* contains germacranolides, seco-germacranolides, furanoheliangolides, and related sesquiterpenoids, with qualitative and quantitative variation depending on plant origin [22,25]. The occurrence of flavonols **C5** and **C6** is consistent with previous reports of methylated 6-hydroxyflavonols in *N. lobata* and related *Neurolaena* species, including *N. oaxacana* [23,24]. Their occurrence in the present study therefore reinforces the recurrence of methylated 6-hydroxyflavonols within the genus *Neurolaena*. In contrast, to the best of our knowledge, the benzoic acid derivatives **C3** and **C4** are reported here for the first time in *N. lobata*. Although hydroxybenzoic acids are widely distributed plant phenolics, their detection expands the known phenolic profile of this medicinal Asteraceae species. From a biological perspective, the isolated metabolites belong to chemical classes of recognized interest in medicinal plant research. The sesquiterpene lactones have been associated with anti-inflammatory, antiproliferative and antiprotozoal bioactivities [22,25,26]. Likewise, methylated flavonols and hydroxybenzoic acid derivatives are widely recognized plant phenolics and have been linked to antioxidants, anti-inflammatory, antimicrobial, and other biological effects [27].

Overall, the isolation of sesquiterpene lactones, benzoic acid derivatives, and methylated flavonols from the selected MeOAc and iPrOH fractions broadens the phytochemical and bioactivity profile of *N. lobata*. These results provide a chemical basis for the subsequent evaluation of the isolated metabolites as potential contributors to the digestive enzyme inhibitory activity observed in the selected fractions.

### 2.3. Determination of Enzyme Inhibition Against PL, AA, and AG

The isolated compounds **C1**–**C6** were evaluated for their inhibitory activity against pancreatic lipase (PL), α-amylase (AA), and α-glucosidase (AG). Half-maximal inhibitory concentration (IC_50_) values were determined for compounds showing quantifiable inhibitory activity. In addition, exploratory kinetic analyses were conducted for the active compounds against PL and AG to obtain apparent inhibition profiles and conditional Ki estimates (Table 2). The benzoic acid derivatives **C3** and **C4** did not show relevant inhibition of any of the three enzymes at the highest concentration tested, indicating that these simple phenolic acids are unlikely to contribute substantially to the digestive enzyme inhibitory activity observed in the selected fractions under the experimental conditions used.

Compounds **C1**, **C2**, **C5** and **C6** inhibited PL, with IC_50_ values ranging from 134.1 to 615.6 µM. Among them, the methylated flavonol **C6** was the most active PL inhibitor, followed by C5, whereas the sesquiterpene lactones **C1** and **C2** showed weaker activity. However, none of the isolated compounds exhibited PL inhibitory activity comparable to that of orlistat, indicating moderate potency relative to the reference inhibitor.

Compounds **C1**, **C2**, **C5** and **C6** also showed measurable inhibition of AG, with IC_50_ values ranging from 170.3 to 639.0 µM. Under the assay conditions used, **C5** and **C6** displayed lower IC_50_ values than acarbose, whereas **C1** and **C2** were less active than the positive control. This numerical comparison should be interpreted cautiously because the assay employed α-glucosidase from *S. cerevisiae*, whose inhibition profile may differ from that of mammalian intestinal α-glucosidases. Further studies using mammalian enzyme models, cellular systems, and in vivo approaches are required to establish the physiological relevance of these findings. In contrast, all isolated compounds showed weak AA inhibition, with inhibition percentages below 15% at the highest concentration tested. This profile indicates that the evaluated sesquiterpene lactones and methylated flavonols do not behave as broad-spectrum digestive enzyme inhibitors but instead show preferential inhibition of PL and AG over AA. To the best of our knowledge, this is the first report evaluating the inhibitory activity of compounds **C1**–**C6** against PL, AG, and AA.

Preliminary structure–activity considerations can be proposed from the inhibition data. Among the flavonols, **C6** showed stronger PL inhibition than **C5**, whereas **C5** was more active than **C6** against AG. Within this structurally related pair, these differences suggest that the additional 3′-hydroxyl group in **C6**, which generates a catechol-type substitution pattern in ring B, may influence inhibitory behavior in an enzyme-dependent manner under the assay conditions used. Likewise, the distinct activities of **C1** and **C2** against PL and AG suggest that differences in their oxidation pattern and α,β-unsaturated carbonyl system may contribute to their inhibitory behavior. Broader conclusions would require evaluation of a larger series of related compounds under comparable assay conditions.

Exploratory kinetic analyses were performed for the active compounds against PL and AG. The apparent kinetic parameters and model-dependent Ki estimates are summarized in Tables S3 and S4, whereas the corresponding Lineweaver–Burk plots are provided in Figures S20 and S21 as supplementary graphical representations. Against PL, **C1**, **C2**, **C5** and **C6** produced concentration-dependent increases in apparent Km with comparatively limited changes in apparent Vmax, yielding profiles consistent with competitive behavior under the assay conditions. A similar pattern was observed for **C1**, **C2** and **C5** against AG. In contrast, **C6** against AG produced relatively limited changes in apparent Km together with a progressive decrease in apparent Vmax as inhibitor concentration increased, consistent with an apparent noncompetitive profile. The apparent Ki estimates ranged from 67.0 to 320 µM. **C6** showed the lowest apparent Ki estimate against PL, whereas **C5** showed the lowest value against AG, in agreement with their respective IC_50_ values. Thus, among the isolated metabolites, the methylated flavonols showed the most favorable inhibitory performance across the two enzyme assays. Because alternative inhibition models were not globally compared, the apparent kinetic profiles and Ki values should be interpreted as model-dependent estimates under the assay conditions rather than as definitive inhibition mechanisms or binding constants.

Overall, comparison of the fraction and pure-compound activities in *N. lobata* indicates that the isolated metabolites did not fully reproduce the inhibitory profile of the selected MeOAc and iPrOH fractions, particularly in the α-amylase assay. Therefore, the activity observed for these fractions should not be attributed exclusively to **C1**–**C6**. Other minor constituents, compositional differences, or matrix-related factors may also contribute to the overall inhibitory profile. Nevertheless, the inhibitory effects observed for **C1**, **C2**, **C5**, and **C6** against PL and AG suggest that sesquiterpene lactones and methylated flavonols may contribute, at least partially, to the activity of the selected fractions from *N. lobata*. However, the weak inhibition of AA, the moderate potency against PL relative to orlistat, and the use of isolated enzyme systems indicate that these compounds should be regarded as digestive enzyme-modulating metabolites rather than potent broad-spectrum inhibitors.

## 3. Materials and Methods

### 3.1. General Experimental Procedures

All commercially available reagents were used without further purification, whereas technical-grade solvents were distilled prior to use. Vacuum liquid chromatography (VLC) was used for fractionation of the ethanolic extract, employing silica gel 60 F_254_ SiliaPlate™ with a particle size of 5–20 µm as stationary phase (SiliCycle^®^ Inc., Quebec City, QC, Canada). Isolation and purification of the chemical constituents were performed by flash chromatography (FC), using silica gel P60 SiliaFlash^®^ with particle sizes of 40–63 µm and/or 20–45 µm as stationary phase (SiliCycle^®^ Inc.). In some cases, preparative thin-layer chromatography (PTLC) was used for purification, employing 1 mm-thick silica gel 60 F_254_ SiliaPlate™ plates (SiliCycle^®^ Inc.). Chromatographic studies and monitoring during fractionation and purification were carried out by thin-layer chromatography (TLC) on silica gel 60 F_254_ SiliaPlate™ plates (SiliCycle^®^ Inc.). Chromatographic homogeneity of the isolated compounds was assessed qualitatively by TLC under UV light at 254 and 365 nm and after visualization with vanillin–sulfuric acid and iodine, together with inspection of the corresponding NMR spectra.

The enzymes used for the enzyme inhibition assays were pancreatic lipase type II (PL) from porcine pancreas (100–500 U/mg protein, EC 3.1.1.3, Sigma-Aldrich, St. Louis, MO, USA), α-amylase type VI-B (AA) from porcine pancreas (≥10 units/mg solid, L-SLBP4061V, EC 3.2.1.1, Sigma-Aldrich), and α-glucosidase type I (AG) from Saccharomyces cerevisiae (lyophilized powder, ≥10 U/mg protein, EC 3.2.1.20, Sigma-Aldrich). The substrates used were 4-nitrophenyl dodecanoate (Sigma-Aldrich, catalog no. 61716) for PL; 2-chloro-4-nitrophenyl-α-D-maltotrioside (CNP-G3; Sigma-Aldrich, catalog no. 93834) for AA; and *p*-nitrophenyl-α-D-glucopyranoside (Sigma-Aldrich, catalog no. N1377) for AG. All other reagents used in the enzymatic assays were of analytical grade, commercially acquired, and used without further treatment. Absorbance readings were performed using a Thermo Scientific Multiskan GO microplate reader (Thermo Fisher Scientific, Waltham, MA, USA) with SkanIt RE 4.1 software.

### 3.2. Plant Material

Leaves of *Neurolaena lobata* (L.) R. Br. ex Cass. (Asteraceae) were collected in January 2019 from a natural population in the surroundings of Quibdó, Chocó Department, Colombia. The plant material was taxonomically identified, and a voucher specimen was deposited at the Herbarium of the University of Antioquia under accession number HUA 205424. The collection and research activities were covered by the amnesty framework established in Article 6 of Colombian Law 1955 of 2019 for activities involving access to genetic resources and their derived products.

### 3.3. Extraction, Fractionation, and Purification

Shade-dried and ground leaves of *N. lobata* (3150 g) were extracted by static maceration at room temperature using EtOH–H_2_O (70:30, *v*/*v*) at a plant material-to-solvent ratio of 1:10 (*w*/*v*). Four consecutive extraction cycles of 48 h each were performed using fresh solvent, for a total extraction time of 8 days. The combined hydroalcoholic extracts were filtered, concentrated under reduced pressure, and lyophilized to obtain the dry hydroalcoholic extract (HA, 100 g; extraction yield: 3.17% *w*/*w*). A portion of the HA extract (96 g) was fractionated by vacuum liquid chromatography (VLC) using solvents of increasing polarity: dichloromethane (DCM, 8.6 g), methyl acetate (MeOAc, 40.2 g), isopropanol (iPrOH, 28.8 g), and an ethanol–water mixture 8:2 (EtOH–H_2_O, 5.3 g) (Figure S1). The resulting fractions were evaluated in vitro against pancreatic lipase (PL), α-amylase (AA) and α-glucosidase (AG). Based on their inhibitory activity, the MeOAc and iPrOH fractions were selected for further phytochemical investigation.

The MeOAc fraction was subjected to flash chromatography (FC) using a hexane–EtOAc gradient system (95:5 to 6:4) as mobile phase, yielding four fractions (Ac1–Ac4). Fraction Ac1 (11.0 g) was purified by successive FC using hexane–EtOAc (6:4) and DCM–acetone (99:1), followed by preparative thin-layer chromatography (PTLC) with toluene–EtOAc (6:4) as the elution system, affording two white amorphous solids corresponding to **C1** (80 mg, m.p. 164–165 °C) and **C2** (50 mg, m.p. 123–124 °C). Fractions Ac2 and Ac3 were combined (10.5 g) and purified by successive FC using hexane–DCM–acetone (5:4.5:0.5), DCM–MeOH (9:1), CHCl_3_–MeOH (9:1), and DCM–acetone (85:15), yielding a yellow solid corresponding to **C3** (21.5 mg, m.p. 206–208 °C). From fraction Ac4 (13.32 g), a yellow solid named **C4** was purified (318.2 mg, m.p. 193–195 °C) by successive FC using DCM–acetone (85:15 to 7:3) and CHCl_3_–EtOAc–MeOH (95:5:5).

The iPrOH fraction (28.8 g) was subjected to FC using a DCM–MeOH gradient system (95:5 to 85:15), yielding three fractions (iPr1–iPr3). Fraction iPr1 (8.06 g) was purified by successive FC using DCM–acetone (99:1) and CHCl_3_–MeOH (9:1), affording a yellow solid corresponding to **C5** (7.8 mg, m.p. 179–181 °C). Fractions iPr2 and iPr3 were combined (14.5 g) and purified by FC using CHCl_3_–MeOH (9:1), yielding a yellow solid named **C6** (22.6 mg, m.p. 108–110 °C). The compounds were identified by one-dimensional NMR (^1^H and APT) and two-dimensional NMR experiments (COSY, HSQC, and HMBC), and by comparison with data reported in the literature.

**Neurolenin B (C1):** white solid, melting point (m.p.): 164–165 °C. ^1^H NMR (400 MHz, CDCl_3_): δ 6.58 (d, *J* = 11.9 Hz, 1H,H-2), 6.30 (s, 1H, H-13a), 6.00 (t, *J* = 11.5 Hz, 1H,H-3), 5.80 (s, 1H,H-13b), 5.54 (s, 2H,H-8,H-9), 4.54 (dd, *J* = 11.8, 4.9 Hz, 1H, H-6), 4.12 (s, 1H, OH), 3.10 (m, 1H, H-4), 2.58 (s, 1H, H-7), 2.09 (s, 1H, H-2′a), 1.95 (s, 3H, H-2′b), 1.94 (m, 1H, H-3′), 1.81 (td, *J* = 13.8, 12.4, 4.9 Hz, 1H, H-5a), 1.41 (td, *J* = 13.8, 11.8, 5.3 Hz, 1H, H-5b), 1.32 (s, 3H, H-14), 1.13 (d, *J* = 6.3 Hz, 3H, H-15), 0.85 (d, *J* = 6.1 Hz, 6H, H-4′,H-5′). APT (100 MHz, CDCl_3_): δ 204.7 (C-1, C=O), 171.1 (C-1′, C=O), 170.3 (C-1¨, C=O), 168.9 (C-12. C=O), 148.3 (CH, C-3), 134.9 (C, C11), 126.6 (CH_2_ C-13), 125.4 (CH, C-2), 79.4 (C, C-10), 76.5 (CH, C-6), 74.0 (CH, C-9), 73.9 (CH, C-8), 42.7 (CH_2_, C-2′), 41.3 (CH, C-7), 40.4 (CH_2_, C-5), 28.3 (CH, C-4), 25.0 (CH, C-3′), 23.8 (CH_3_, C-14), 22.5 (CH_3_, C-4′), 22.3 (CH_3_, C-5′), 20.7 (CH_3_, C-2″), 19.8 (CH_3_, C-15). The spectroscopic data were consistent with those reported in the literature for neurolenin B’ [22]. The spectroscopic data can be consulted in Figures S2–S6 and Table S1 in Supplementary Materials.
**Lobatin A (C2):** white solid, melting point (m.p.): 123–124 °C. ^1^H NMR (400 MHz, CDCl_3_): δ 6.31 (d, *J* = 3.4 Hz, 1H, H-13a), 5.91 (t, *J* = 9.58, 8.13 Hz, 1H, H-3), 5.85 (dd, *J* = 10.4, 1.6 Hz, 1H, H-8), 5.69 (d, *J* = 2.9 Hz, 1H,H-13b), 5.65 (d, *J* = 10.4 Hz, 1H, H-9), 4.92 (dt, *J* = 7.7, 3.8 Hz, 1H, H-6), 4.25 (s, 1H, OH), 3.57 (dd, *J* = 15.9, 9.6 Hz, 1H, H-2a), 3.06 (dd, *J* = 15.9, 7.9 Hz, 1H, H-2b), 2.82 (dd, *J* = 15.1, 3.6 Hz, 1H, H-5a), 2.73 (dd, *J* = 15.0, 4.2 Hz, 1H, H-5b), 2.61 (m, *J* = 7.8, 3.1, 1.6 Hz, 1H, H-7), 2.14 (s, 1H, H-2″), 2.12 (m, 1H, H-2′a), 2.08 (m, 1H, H-2′b), 1.84 (s, 3H,H-15), 1.33 (s, 3H,H-14), 0.88 (d, *J* = 4.4 Hz, 3H, H-4′), 0.85 (d, *J* = 4.5 Hz, 3H, H-5′). APT (100 MHz, CDCl_3_): δ 210.1 (C, C=O), 171.0 (C, C=O), 170.5 (C, C=O-1′), 168.2 (C, C=O), 136.8 (C, C-4), 134.4 (C, C-11), 124.4 (CH_2_, C-13), 121.5 (CH, C-3), 80.6 (C, C-10), 76.7 (CH, C-9), 72.6 (CH, C-6), 76.6 (CH, C-8), 42.9 (CH_2_, C-5), 42.2 (CH, C-7), 36.2 (CH_2_, C-2′), 42.1 (CH_2_, C-2), 25.6 (CH_3_, C-14), 25.5(CH, C-3′), 22.6 (CH_3_, C-15), 22.5 (CH_3_, C-4′), 22.4 (CH_3_, C-5′), 20.7 (CH_3_, C-2″). The spectroscopic data were consistent with those reported in the literature for lobatin A [22]. The spectroscopic data can be consulted in Figures S8–S11 and Table S2 in Supplementary Materials.
**p-Hydroxybenzoic acid (C3)**: yellow solid, melting point (m.p.): 206–208 °C. ^1^H NMR (400 MHz, acetone-*d*_6_): δ 7.92 (d, *J* = 8.8 Hz, 2H, H-2, H-6), 6.92 (d, *J* = 8.8 Hz, 2H, H-3, H-5). APT (100 MHz, acetone-*d*_6_): δ 115.9 (CH, C-3, 5), 122.6 (C, C-1), 132.7 (CH, C-2, 6), 162.6 (C, C-4), 167.7 (C, C=O). The spectroscopic data were consistent with those reported in the literature for p-hydroxybenzoic acid [28]. The spectroscopic data can be consulted in Figures S12 and S13 in Supplementary Materials.
**3,4-Dihydroxybenzoic acid (C4):** yellow solid, melting point (m.p.): 193–195 °C. ^1^H NMR (400 MHz, CD_3_OD): δ 7.53 (d, *J* = 2.0 Hz, 1H, H-2), 7.47 (dd, *J* = 8.3, 2.1 Hz, 1H, H-6), 6.90 (d, *J* = 8.3 Hz, 1H, H-5), APT (100 MHz, CD_3_OD): δ 115.7 (CH, C-5), 117.7 (CH, C-2), 123.5 (C, C-1), 123.9 (CH, C-6), 146.0 (C, C-3), 151.5 (C, C-4), 170.2 (C, C=O). The spectroscopic data were consistent with those reported in the literature for 3,4-dihydroxybenzoic acid [29]. The spectroscopic data can be consulted in Figures S14 and S15 in Supplementary Materials.
**6-Hydroxykaempferol 3,7-dimethyl ether (C5)**: yellow solid, melting point (m.p.): 179–181 °C. ^1^H NMR: (400 MHz, acetone-*d*_6_) δ 12.48 (s, 1H), 8.03 (d, *J* = 8.9 Hz, 2H, H-2′, H-6′), 7.01 (d, *J* = 8.9 Hz, 2H, H-3′, H-5′), 6.81 (s, 1H, H-8), 3.97 (s, 3H, H-1″), 3.86 (s, 3H, H-2″). APT (100 MHz, acetone-*d*_6_): δ 179.6 (C, C=O), 160.8 (C, C-4′), 156.9 (C, C-7), 150.3 (C, C-9), 146.8 (C, C-5), 157.1 (C, C-2), 138.1 (C, C-3), 130.1 (C, C-6), 130.8 (CH, C-2′,6′), 122.7 (C, C-1′), 116.3 (CH, C -3′,5′), 106.9 (C, C-10), 91.2 (CH, C-8), 60.1 (CH_3_, C-2″), 56.7 (CH_3_, C-1″). The spectroscopic data were consistent with those reported in the literature for 6-hydroxykaempferol 3,7-dimethyl ether [23]. The spectroscopic data can be consulted in Figures S16 and S17 in Supplementary Materials.
**Quercetagetin 3,7-dimethyl ether (C6):** yellow solid, melting point (m.p.): 108–110 °C. ^1^H NMR (400 MHz, acetone-*d*_6_): δ 12.47 (s, 1H-5-OH), 7.71 (d, *J* = 2.2 Hz, 1H-2′), 7.58 (dd, *J* = 8.5, 2.2 Hz, 1H-6′), 6.99 (d, *J* = 8.5 Hz, 1H-5′), 6.80 (s, 1H-8), 3.97 (s, 3H-1″,3-OCH_3_), 3.86 (s, 3H-2″, 7-OCH_3_). APT (100 MHz, acetone-*d*_6_): δ 179.6 (C, C=O), 150.8 (C, C-9), 154.9 (C, C-7), 146.8 (C, C-5), 149.0 (C, C-2), 146.7 (C, C-3′), 145.9 (C, C-4′), 139.0 (C, C-3), 130.7 (C, C-6), 123.1 (C, C-1′), 122.0 (CH, C-6′), 116.3 (CH, C-5′), 116.2 (CH, C-2′), 106.8 (C, C-10), 91.2 (CH, C-8), 60.1 (CH_3_, C-1″,3-OCH_3_), 56.7 (CH_3_, C-2″,7-OCH_3_). The spectroscopic data were consistent with those reported in the literature for quercetagetin 3,7-dimethyl ether [23]. The spectroscopic data can be consulted in Figures S18 and S19 in Supplementary Materials.

### 3.4. Enzyme Inhibition Assays Against PL, AA, and AG

The inhibitory activity of the hydroalcoholic extract, VLC fractions, and isolated compounds against pancreatic lipase (PL), α-amylase (AA), and α-glucosidase (AG) was evaluated using endpoint spectrophotometric microplate assays. Absorbance was measured at 405 nm after 25 min for PL and AG and after 30 min for AA. These incubation times were selected from preliminary time-course experiments showing that the assays remained within the linear reaction range.

Extracts and fractions were evaluated at concentrations ranging from 1000 to 62.5 µg/mL and from 500 to 6.25 µg/mL, respectively. Isolated compounds were initially screened at a final concentration of 300 µM. Compounds producing inhibition close to or above 50% at this concentration were subsequently evaluated in concentration–response assays at final concentrations in the reaction wells ranging from 9.37 to 1000 µM, as required to adequately bracket the IC_50_ value. IC_50_ values were estimated by nonlinear regression only when 50% inhibition was reached within the experimentally tested range. All test samples were prepared as DMSO stock solutions and diluted in the corresponding assay buffer. The final DMSO concentration was kept constant across all control and sample wells and was 5% (*v*/*v*) in the PL and AA assays and 4% (*v*/*v*) in the AG assay; these concentrations did not significantly affect enzyme activity.

Sample blanks containing test sample and substrate without enzyme were included to correct for sample background absorbance and nonenzymatic substrate hydrolysis. Enzyme-free control blanks containing substrate and vehicle without enzyme were used to correct for spontaneous substrate hydrolysis, whereas substrate-free wells were included to monitor baseline absorbance. Vehicle controls contained enzyme, substrate, and the corresponding DMSO concentration without test sample. Absorbance values were used to calculate relative enzyme activity and percentage inhibition. Inhibition percentage was calculated as:

$$\%Inhibition=\left\lfloor 1-\frac{\left(A_{sample}-A_{sample\;blank}\right)}{\left(A_{vehicle\;control}-A_{control\;blank}\right)}\right\rfloor \times 100$$

where *A_sample_* is the absorbance of wells containing enzyme, substrate and test sample; *A_sample blank_* is the absorbance of the corresponding enzyme-free wells containing sample and substrate; *A_vehicle control_* is the absorbance of wells containing enzyme, substrate, and the corresponding DMSO concentration without test sample; and *A_control blank_* is the absorbance of enzyme-free wells containing substrate and vehicle. All experiments were performed in triplicate in three independent experiments. IC_50_ values were estimated by nonlinear regression analysis using GraphPad Prism 8.

#### 3.4.1. PL Inhibition Assay

PL inhibitory activity was evaluated according to the method proposed in previous reports [6,30], with some modifications. In 96-well plates, 30 µL of PL working solution, prepared in 50 mM Tris-HCl buffer (pH 8.5) containing 5 mM sodium deoxycholate (SDC) and 5 mM NaCl, was added to obtain a final enzyme concentration of 1 mg/mL in the 250 µL reaction mixture. The enzyme suspension was centrifuged at 5000 rpm for 10 min to remove suspended particles and pre-incubated at 37 °C for 5 min. Subsequently, 30 µL of the test-sample working solution and 160 µL of 50 mM Tris-HCl buffer (pH 8.5) were added. The reaction was initiated by adding 30 µL of p-nitrophenyl dodecanoate to obtain a final substrate concentration of 0.075 mM. Test samples were evaluated at the final concentrations indicated for each assay. After incubation for 25 min at 37 °C, absorbance was measured at 405 nm. Orlistat was used as the positive control. Test samples and orlistat were evaluated at the indicated final concentrations in the reaction wells.

#### 3.4.2. AG Inhibition Assay

AG inhibitory activity was evaluated according to the method proposed in previous reports [31,32], with some modifications. In 96-well plates, 20 µL of an AG working solution (6.25 U/mL), prepared in 50 mM sodium phosphate buffer (pH 6.4) containing 5 mM NaCl, was added to obtain a final enzyme activity of 0.50 U/mL in the 250 µL reaction mixture. The enzyme solution was pre-incubated at 37 °C for 10 min, followed by the addition of 10 µL of test-sample working solution and 200 µL of sodium phosphate buffer (50 mM, pH 6.4, containing 5 mM NaCl). The reaction was initiated by adding 10 µL of p-nitrophenyl α-D-glucopyranoside working solution (2.75 mM), yielding a final substrate concentration of 110 µM. The reaction mixture was incubated for 25 min at 37 °C, and absorbance was measured at 405 nm. Acarbose was used as the positive control. Test samples and acarbose were evaluated at the indicated final concentrations in the reaction wells.

#### 3.4.3. AA Inhibition Assay

AA inhibitory activity was evaluated according to the method proposed in previous reports [33,34,35,36], with some modifications. In 96-well plates, 30 µL of an AA working solution (16.67 U/mL), prepared in 50 mM sodium phosphate buffer (pH 6.4) containing 5 mM NaCl, was added to obtain a final enzyme activity of 2.0 U/mL in the 250 µL reaction mixture. The enzyme solution was pre-incubated at 37 °C for 10 min, followed by the addition of 30 µL of test-sample working solution and 160 µL of sodium phosphate buffer (50 mM, pH 6.4, containing 5 mM NaCl). The reaction was initiated by adding 30 µL of 2-chloro-4-nitrophenyl-α-D-maltoside working solution (4.443 mM), yielding a final substrate concentration of 533.2 µM. The reaction mixture was incubated for 30 min at 37 °C, and absorbance was measured at 405 nm. Test samples and acarbose were evaluated at the indicated final concentrations in the reaction wells. The final DMSO concentration was 5% (*v*/*v*) and was maintained constant in all sample and control wells. Acarbose was used as the positive control.

### 3.5. Exploratory Kinetic Analysis

Exploratory kinetic analyses were performed for the active compounds against PL and AG under the endpoint assay conditions described above. All substrate and inhibitor concentrations refer to final concentrations in the 250 µL reaction wells. For PL, substrate concentrations of 15.6, 31.3, 62.5, 125, 250, 500 and 1000 µM were evaluated. For AG, substrate concentrations of 78.1, 156.3, 312.5, 625, 1250, 2500, and 5000 µM were evaluated. Active compounds were tested at final concentrations corresponding to 0.5, 1.0, and 2.0× their respective IC_50_ values, together with an uninhibited control; the exact inhibitor concentrations are provided in Tables S3 and S4. Substrate–response curves at each inhibitor level were individually fitted to obtain apparent Km and Vmax values. The complete datasets comprising all substrate and inhibitor concentrations for each compound–enzyme pair were subsequently entered into GraphPad Prism 8 for global nonlinear regression using the competitive or noncompetitive inhibition model corresponding to the apparent profile assigned from the kinetic trends and Lineweaver–Burk plots (Figures S20 and S21). Because alternative inhibition models were not formally compared, the resulting profiles and Ki values are described as apparent, model-dependent estimates under the assay conditions.

### 3.6. Statistical Analysis

Data were analyzed using GraphPad Prism 8. Each condition was tested in technical triplicate across three independent experiments (n = 3); technical replicates were averaged within each experiment. IC_50_ values are reported as mean ± SD and were estimated by nonlinear regression. Apparent Ki values were obtained by global nonlinear regression using the complete substrate–inhibitor dataset and are reported as model-dependent exploratory estimates.

## 4. Conclusions

The hydroalcoholic extract of *N. lobata* leaves and the selected MeOAc and iPrOH fractions showed in vitro inhibitory activity against pancreatic lipase (PL), α-glucosidase (AG), and α-amylase (AA). Among the isolated metabolites, neurolenin B (**C1**), lobatin A (**C2**), 6-hydroxykaempferol 3,7-dimethyl ether (**C5**) and quercetagetin 3,7-dimethyl ether (**C6**) showed measurable inhibition of PL and AG, whereas their activity against AA was weak under the experimental conditions used. None of the isolated compounds showed PL inhibition comparable to that of orlistat, indicating moderate potency in this assay. The isolated metabolites did not fully reproduce the inhibitory profile of the active fractions, particularly against AA. Overall, these results expand the phytochemical and in vitro bioactivity profile of *N. lobata* and indicate that sesquiterpene lactones and methylated flavonols may contribute, at least partially, to the digestive enzyme-modulating activity of its leaf fractions. However, the weak inhibition of AA, the moderate potency against PL relative to orlistat, and the use of α-glucosidase from *S. cerevisiae* limit the biological interpretation of these findings. Therefore, the results should be regarded as preliminary in vitro evidence of enzyme modulation and do not establish antidiabetic, anti-obesity, or therapeutic efficacy. Further studies using mammalian enzyme models, cellular systems, bioavailability assessment, and in vivo approaches are required to establish physiological relevance.

## Figures and Tables

**Table 1:** Half-maximal inhibitory concentrations (IC_50_) obtained for the HA extract and fractions from *N. lobata* leaves.

Samples/Controls (+)	PL IC_50_ μg/mL	AA IC_50_ μg/mL	AG IC_50_ μg/mL
HA	166.3 ± 1.8	202.0 ± 1.1	74.8 ± 2.3
DCM	NA (13.2%)	NA (5.6%)	NA (48.5%)
MeOAc	317.9 ± 3.1	141.2 ± 2.1	94.4 ± 3.9
iPrOH	251.1 ± 0.6	691.1 ± 3.6	78.5 ± 3.0
EtOH-H_2_O	NA (21.5%)	NA (6.3%)	NA (38.1%)
Orlistat	0.30 ± 0.10	ND	ND
Acarbose	ND	2.04 ± 1.68	223.3 ± 2.1

PL: pancreatic lipase; AA: α-amylase; AG: α-glucosidase; HA: hydroalcoholic extract; DCM: dichloromethane; MeOAc: methyl acetate; iPrOH: isopropanol; EtOH–H_2_O: ethanol–water; the results are reported as the mean ± SD (standard deviation). NA: IC_50_ could not be determined within the tested concentration range; ND: not determined. Values in parentheses indicate the percentage inhibition at the highest concentration tested (1000 µg/mL for the HA extract and 500 µg/mL for the VLC fractions).

**Table 2:** In vitro inhibitory activity and apparent kinetic profiles of compounds **C1**–**C6** against PL, AG and AA.

Compound/Control (+)	Pancreatic Lipase (PL)			α-Glucosidase (AG)			α-Amylase (AA)		
	IC_50_ (µM)	Apparent Ki Estimate (µM) *	Apparent Kinetic Profile	IC_50_ (µM)	Apparent Ki Estimate (µM) *	Apparent Kinetic Profile	IC_50_ (µM)	Apparent Ki Estimate (µM) *	Apparent Kinetic Profile
**C1**	591.8 ± 1.2	285.5	Consistent with competitive behavior	639.0 ± 3.8	318.2	Consistent with competitive behavior	NA (7.8%)	ND	ND
**C2**	615.6 ± 2.9	305.5	Consistent with competitive behavior	525.1 ± 4.7	238.7	Consistent with competitive behavior	NA (9.5%)	ND	ND
**C3**	NA (37.8%)	ND	ND	NA (39.1%)	ND	ND	NA (4.5%)	ND	ND
**C4**	NA (31.2%)	ND	ND	NA (47.3%)	ND	ND	NA (2.4%)	ND	ND
**C5**	166.4 ± 3.2	83.2	Consistent with competitive behavior	170.3 ± 2.2	85.0	Consistent with competitive behavior	NA (13.8%)	ND	ND
**C6**	134.1 ± 0.7	67.0	Consistent with competitive behavior	215.2 ± 2.1	107.2	Consistent with noncompetitive behavior	NA (12.5%)	ND	ND
**Orlistat**	0.6 ± 0.2	1.7	Irreversible	ND	ND	ND	ND	ND	ND
**Acarbose**	ND	ND	ND	345.9 ± 3.2	56.3	Consistent with competitive behavior	3.16 ± 2.6	35.5	Consistent with competitive behavior

IC_50_ values are expressed as mean ± SD from three independent experiments (*n* = 3), each performed in technical triplicate, for a total of nine technical wells per concentration. NA: inhibition < 50% at the initial screening concentration; values in parentheses indicate percentage inhibition at 300 µM. ND: not determined. * Apparent kinetic profiles and Ki estimates should be interpreted as model-dependent results under the assay conditions. Full apparent kinetic parameters are provided in Tables S3 and S4.

## Data Availability

The original contributions presented in this study are included in the article. Further inquiries can be directed at the corresponding author.

## Supplementary Materials

The following supporting information can be downloaded at: https://www.mdpi.com/article/10.3390/molecules31152596/s1, Figure S1: Schematic Isolation of compounds from *Neurolaena lobata* (L.) R. Br. ex Cass.—Asteraceae; Figure S2: ^1^H-NMR spectra of compound Neurolenin B (C1), (400 MHz, CDCl_3_); Figure S3: APT spectra of Neurolenin B (C1), (100 MHz, CDCl_3_); Figure S4: COSY spectra of Neurolenin B (C1); Figure S5: HSQC spectra of Neurolenin B (C1); Figure S6: HMBC spectra of Neurolenin B (C1); Figure S7: ^1^H-NMR spectra of Lobatin A (C2), (400 MHz, CDCl_3_); Figure S8: APT spectra of Lobatin A (C2), (100 MHz, CDCl_3_); Figure S9: COSY spectra of Lobatin A (C2); Figure S10: HSQC spectra of Lobatin A (C2); Figure S11: HMBC spectra of Lobatin A (C2); Figure S12: ^1^H-NMR spectra of p-Hydroxybenzoic acid (C3), (400 MHz, acetone-*d*_6_); Figure S13: APT spectra of p-Hydroxybenzoic acid (C3), (100 MHz, acetone-*d*_6_); Figure S14: ^1^H-NMR spectra of 3,4-Dihydroxybenzoic acid (C4), (400 MHz, CD_3_OD); Figure S15: APT spectra of 3,4-Dihydroxybenzoic acid (C4), (100 MHz, CD_3_OD); Figure S16: ^1^H-NMR spectra of 6-Hydroxykaempferol 3,7-dimethyl ether (C5), (400 MHz, acetone-*d*_6_); Figure S17: APT spectra of 6-Hydroxykaempferol 3,7-dimethyl ether (C5), (100 MHz, acetone-*d*_6_); Figure S18: ^1^H-NMR spectra of Quercetagetin 3,7-dimethyl ether (C6), (400 MHz, acetone-*d*_6_); Figure S19: APT spectra of Quercetagetin 3,7-dimethyl ether (C6), (100 MHz, acetone-*d*_6_); Figure S20: Lineweaver–Burk plots for apparent inhibition profiles on α-glucosidase (AG) by C1, C2, C5 and C6; Figure S21: Lineweaver–Burk plots for apparent inhibition profiles on pancreatic lipase (PL) by C1, C2, C5 and C6; Table S1: ^1^H and ^13^C NMR spectroscopic data of Neurolenin B (C1) and comparison with literature values; Table S2: ^1^H and ^13^C NMR spectroscopic data of Lobatin A (C2) and comparison with literature values; Table S3: Apparent Michaelis–Menten parameters and Ki estimates for the inhibition of pancreatic lipase (PL) by C1, C2, C5, and C6; Table S4: Apparent Michaelis–Menten parameters and Ki estimates for the inhibition of α-glucosidase (AG) by C1, C2, C5, and C6.
