# Supplementary material for: In Vitro Inhibition of Digestive Enzymes by Leaf Metabolites from Neurolaena lobata (L.) R. Br. ex Cass. (Asteraceae)
Source: Molecules. 2026 Jul 25;31(15):2596. doi: 10.3390/molecules31152596 (PMC13468030; doi:10.3390/molecules31152596)
Supplement: Supplementary file 1 [file molecules-31-02596-s001.zip › molecules-4353878-supplementary.pdf]

molecules

In Vitro Inhibition of Digestive Enzymes by Leaf Metabolites from *Neurolaena lobata* (L.) R. Br. ex Cass. (Asteraceae)

Yohum lozada-Diaz [1,2], Oscar J. Patiño-Ladino [2] and Juliet A. Prieto-Rodríguez [1,*]

Supplementary materials

Table of content

Figure S1. Schematic Isolation of compounds from *Neurolaena lobata* (L.) R. Br. ex Cass. – Asteraceae.
Figure S2. ^{1}H-NMR spectrum of compound Neurolenin B (**C1**), (400 MHz, $CDCl_3$).
Figure S3. APT spectrum of Neurolenin B (**C1**), (100 MHz, $CDCl_3$).
Figure S4. COSY spectra of Neurolenin B (**C1**).
Figure S5. HSQC spectra of Neurolenin B (**C1**).
Figure S6. HMBC spectra of Neurolenin B (**C1**).
Table S1. ^{1}H and ^{13}C NMR spectroscopic data of Neurolenin B (**C1**) and comparison with literature values.
Figure S7. ^{1}H-NMR spectrum of Lobatin A (**C2**), (400 MHz, $CDCl_3$).
Figure S8. APT spectrum of Lobatin A (**C2**), (100 MHz, $CDCl_3$).
Figure S9. COSY spectra of Lobatin A (**C2**).
Figure S10. HSQC spectra of Lobatin A (**C2**).
Figure S11. HMBC spectra of Lobatin A (**C2**).
Table S2. ^{1}H and ^{13}C NMR spectroscopic data of Lobatin A (C2) and comparison with literature values.
Figure S12. ^{1}H-NMR spectrum of p-Hydroxybenzoic acid (**C3**), (400 MHz, acetone-d_6).
Figure S13. APT spectrum of p-Hydroxybenzoic acid (**C3**), (100 MHz, acetone-d_6).
Figure S14. ^{1}H-NMR spectrum of 3,4-Dihydroxybenzoic acid (**C4**), (400 MHz, CD_3OD).
Figure S15. APT spectrum of 3,4-Dihydroxybenzoic acid (**C4**), (100 MHz, CD_3OD).
Figure S16. ^{1}H-NMR spectrum of 6-Hydroxykaempferol 3,7-dimethyl ether (**C5**), (400 MHz, acetone-d_6).
Figure S17. APT spectrum of 6-Hydroxykaempferol 3,7-dimethyl ether (**C5**), (100 MHz, acetone-d_6).
Figure S18. ^{1}H-NMR spectrum of Quercetagetin 3,7-dimethyl ether (**C6**), (400 MHz, acetone-d_6).
Figure S19. APT spectrum of Quercetagetin 3,7-dimethyl ether (**C6**), (100 MHz, acetone-d_6).
Figure S20. Lineweaver–Burk plots for apparent inhibition profiles on α-glucosidase (AG) by **C1**, **C2**, **C5** and **C6.**
Figure S21. Lineweaver–Burk plots for apparent inhibition profiles on pancreatic lipase (PL) by **C1**, **C2**, **C5** and **C6.**
Table S3. Apparent Michaelis–Menten parameters and Ki estimates for the inhibition of pancreatic lipase (PL) by **C1**, **C2**, **C5** and **C6**.
Table S4. Apparent Michaelis–Menten parameters and Ki estimates for the inhibition of α-glucosidase (AG) by **C1**, **C2**, **C5** and **C6**.

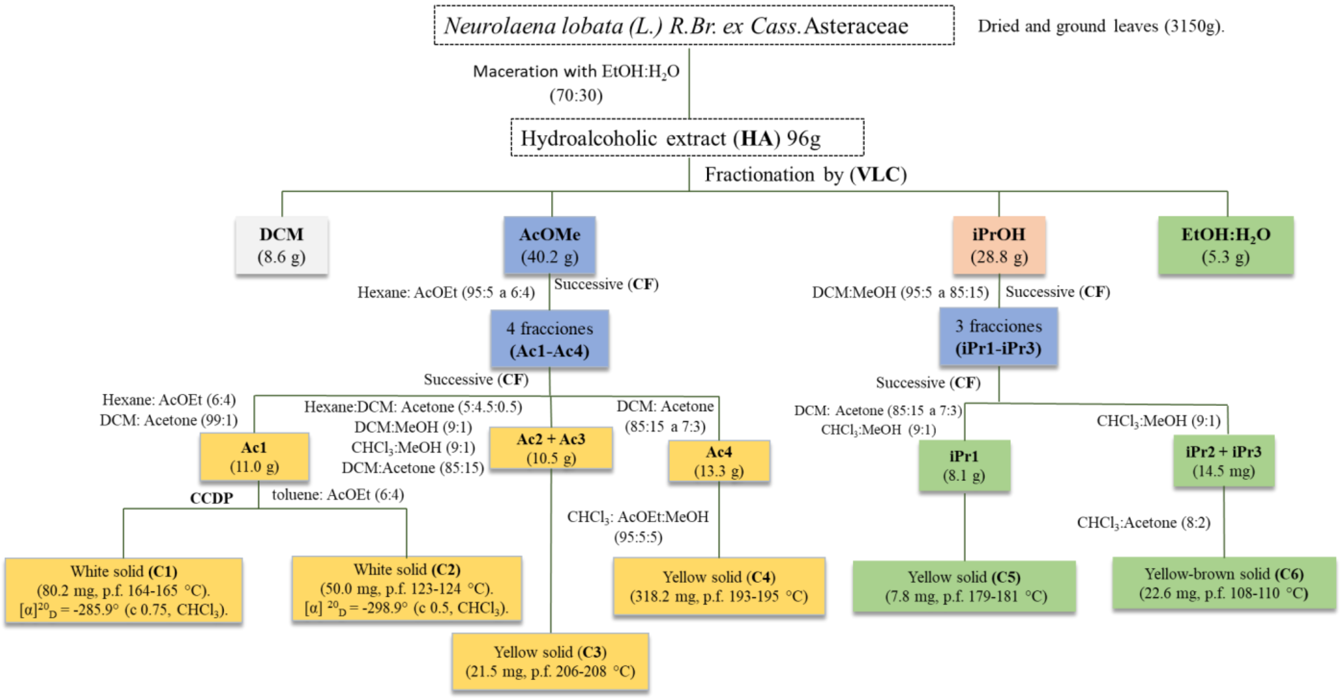

Figure S1. Schematic Isolation of compounds from *Neurolaena lobata* (L.) R. Br. ex Cass. - Asteraceae.

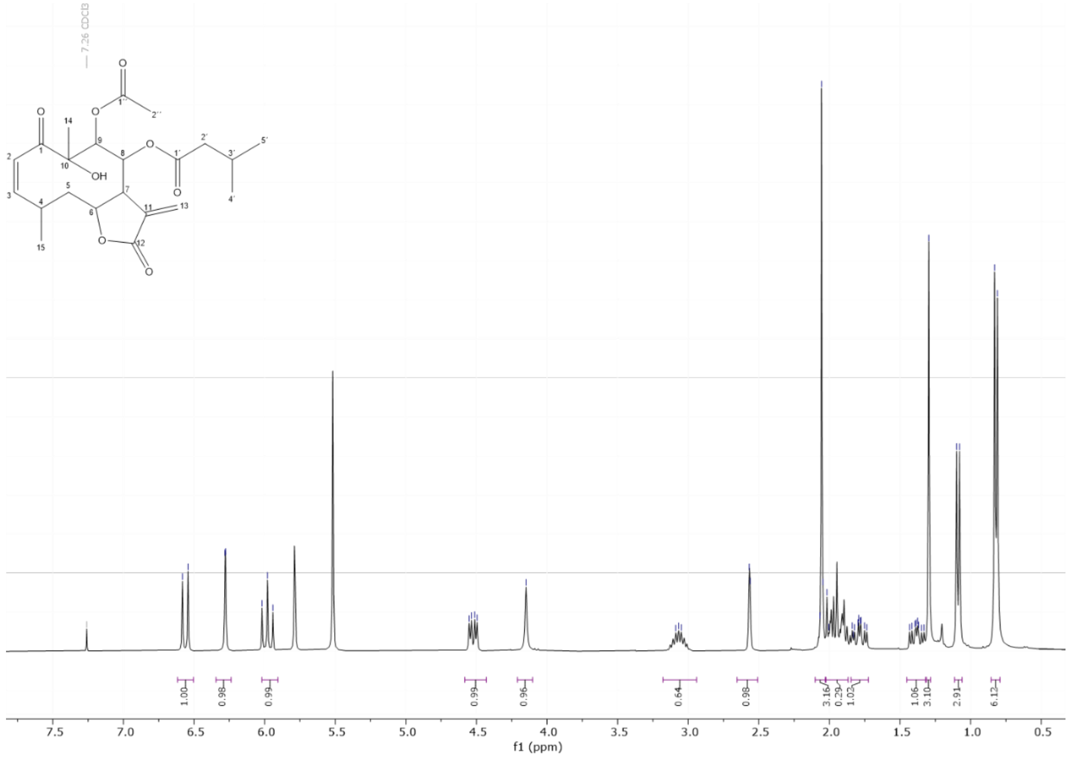

Figure S2. ^{1}H-NMR spectrum of compound Neurolenin B (**C1**), (400 MHz, $CDCl_3$).

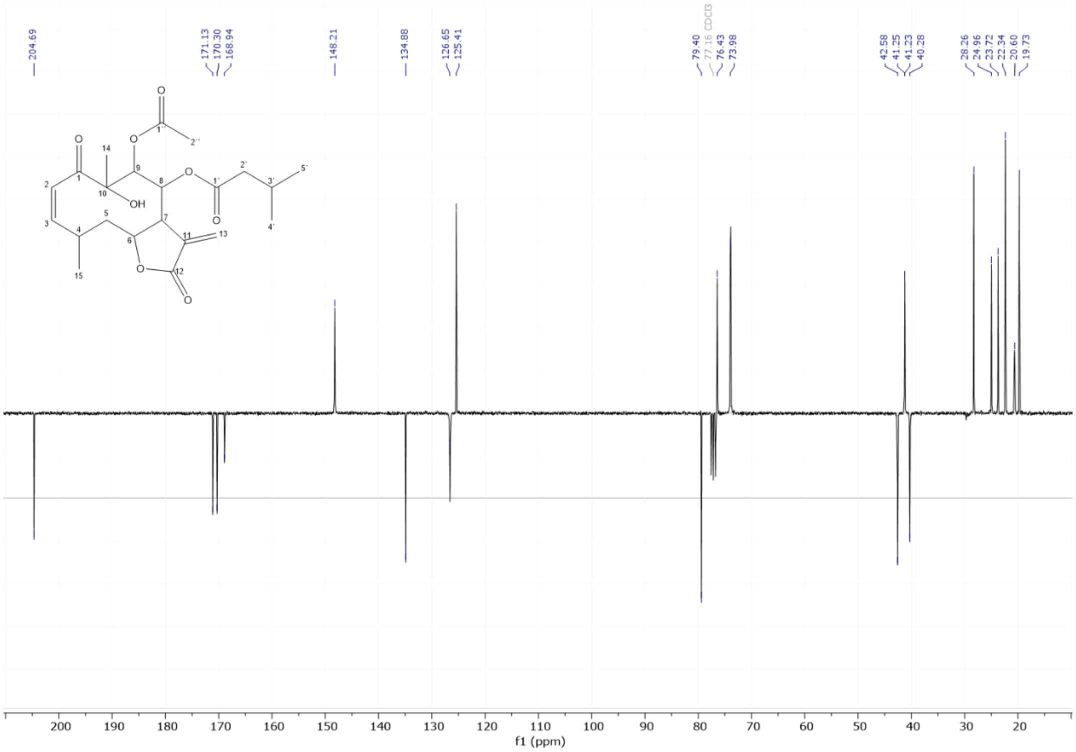

Figure S3. APT spectrum of Neurolenin B (**C1**), (100 MHz, $CDCl_3$).

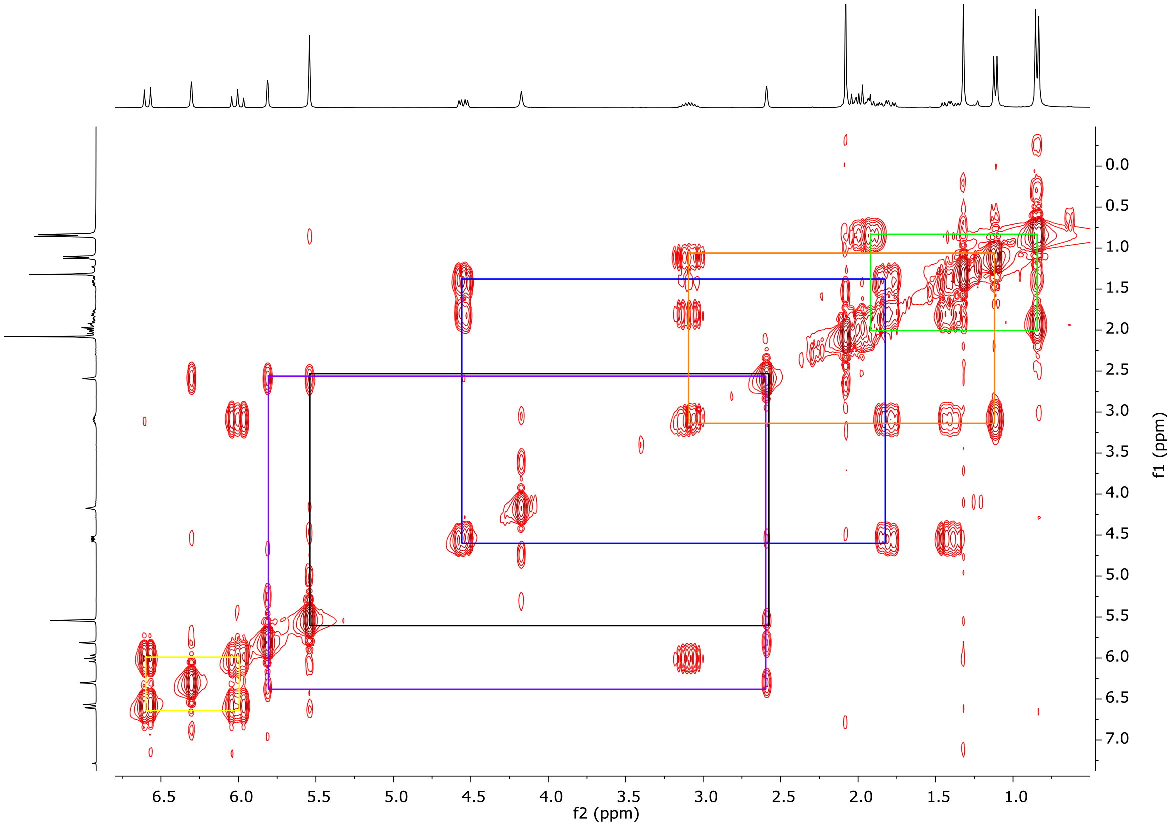

Figure S4. COSY spectra of Neurolenin B (**C1**).

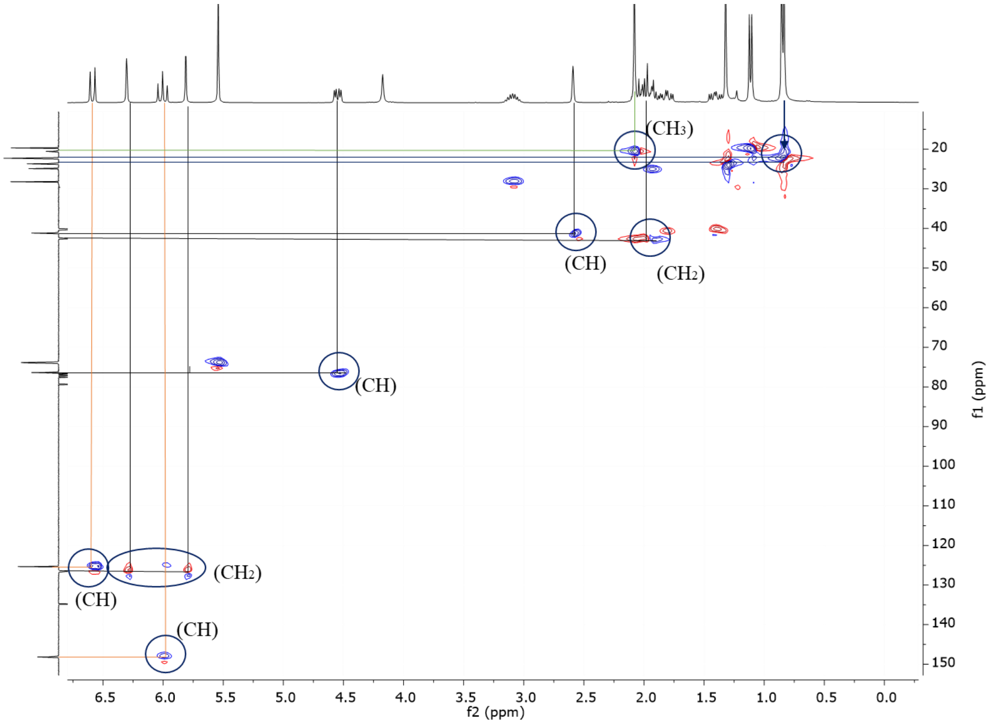

Figure S5. HSQC spectra of Neurolenin B (**C1**).

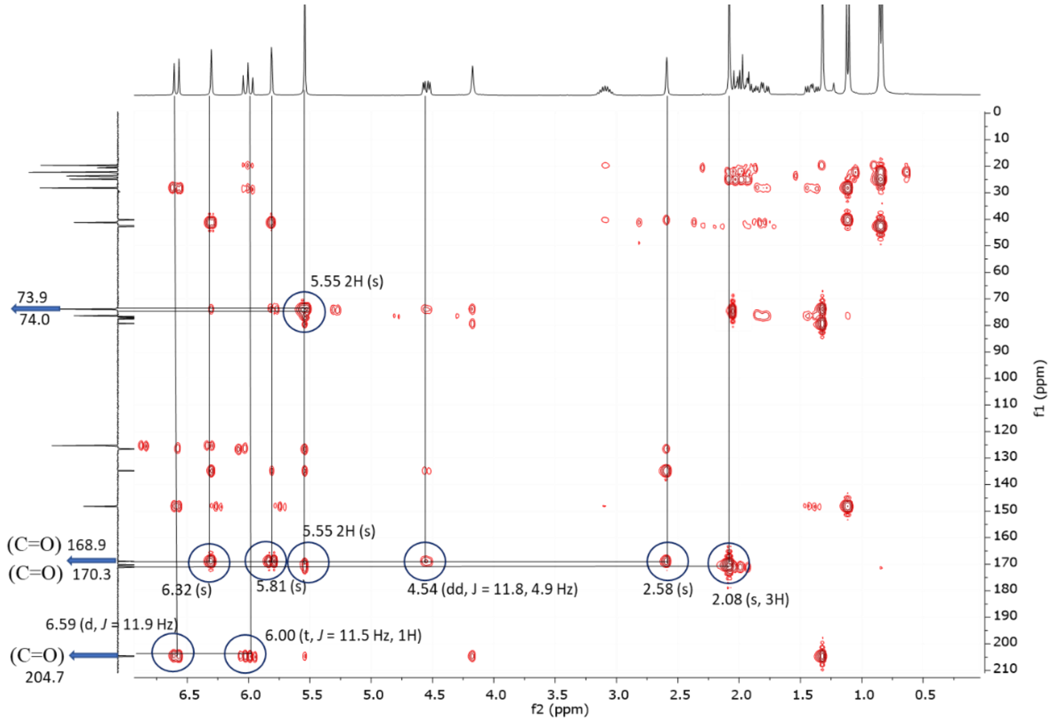

Figure S6. HMBC spectra of Neurolenin B (**C1**).

molecules

Table S1. ^{1}H and ^{13}C NMR spectroscopic data of Neurolenin B (**C1**) and comparison with literature values.

**Signal**	**Observed δ**		**Literature δ [22]**	
	**^{1}H NMR**	**^{13}C NMR**	**^{1}H NMR**	**^{13}C NMR**
**1**	-	204.7	-	204.7
**2**	6.58	125.4	6.59	125.4
**3**	6.00	148.3	6.00	148.2
**4**	3.10	28.3	3.11	28.3
**5a**	1.81	40.4	1.83	40.3
**5b**	1.41		1.42	
**6**	4.54	76.5	4.56	76.4
**7**	2.58	41.3	2.58	41.3
**8**	5.54	73.9	5.55	74.0
**9**	5.54	74.0	5.55	73.9
**10**	-	79.4	-	79.3
**11**	-	134.9	-	134.9
**12**	-	168.9	-	168.7
**13a**	6.30	126.6	6.31	126.5
**13b**	5.80		5.81	
**14**	1.32	23.8	1.33	23.7
**15**	1.13	19.8	1.14	19.7
**1´**	-	171.1	-	171.1
**2´a**	2.09	42.7	2.09	42.6
**2´b**	1.95		1.96	
**3´**	1.94	25.0	1.95	24.9
**4´**	0.85	22.5	0.87	22.3
**5´**	0.85	22.3	0.87	22.3
**1´´**	-	170.3	-	170.3
**2´´**	2.09	20.7	2.10	20.6

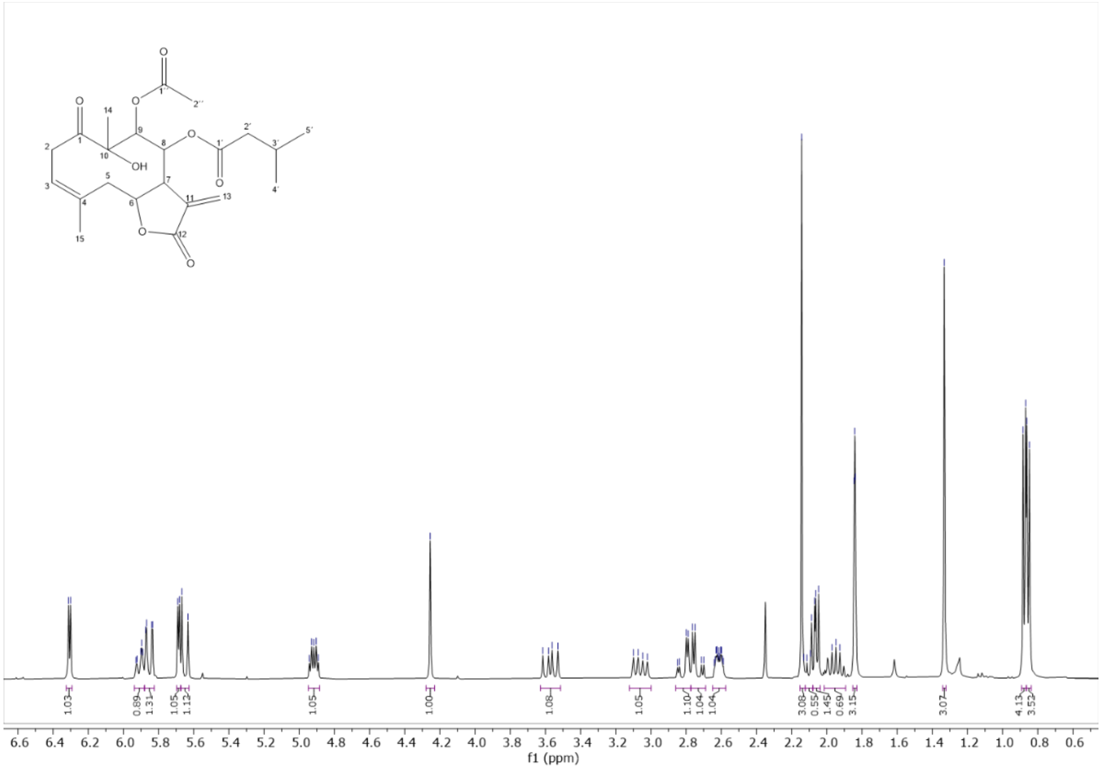

Figure S7. ^{1}H-NMR spectrum of Lobatin A (**C2**), (400 MHz, $CDCl_3$).

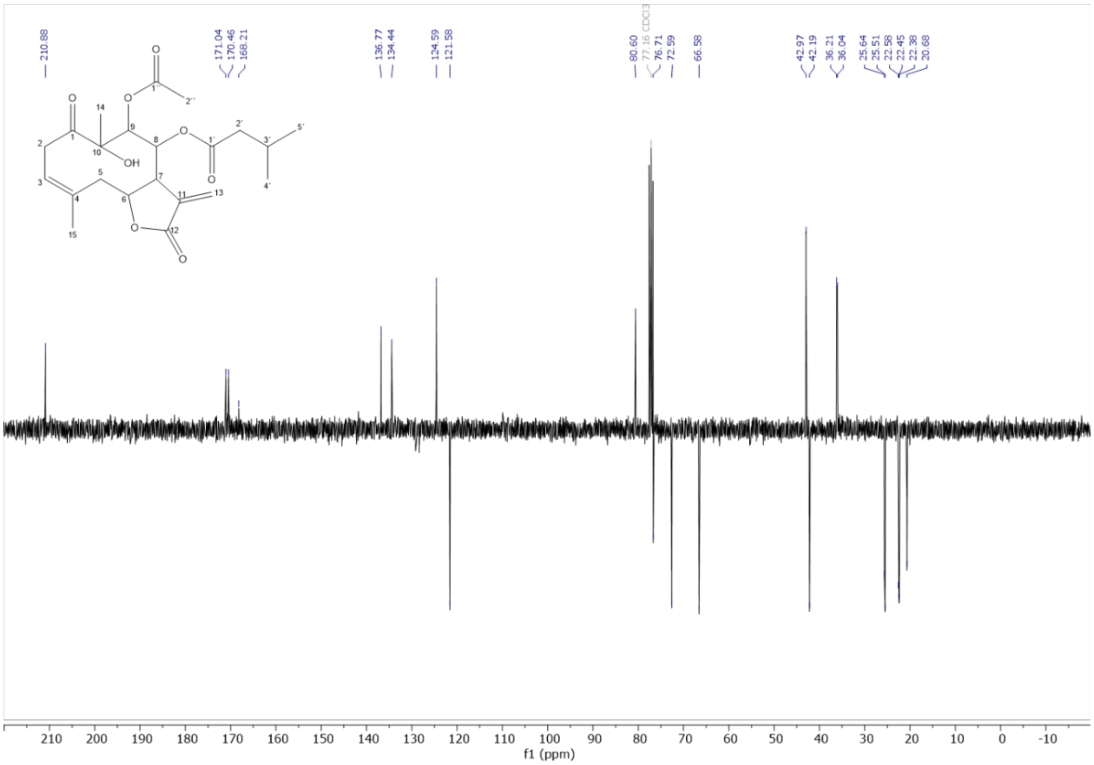

Figure S8. APT spectrum of Lobatin A (**C2**), (100 MHz, $CDCl_3$).

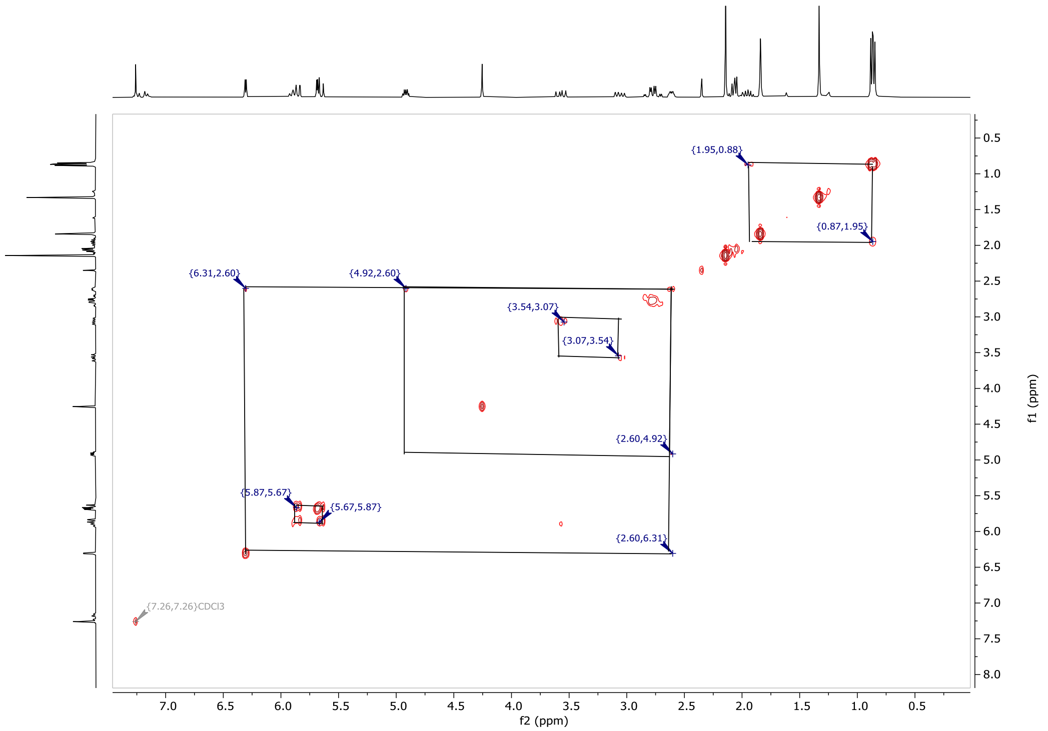

Figure S9. COSY spectra of Lobatin A (**C2**).

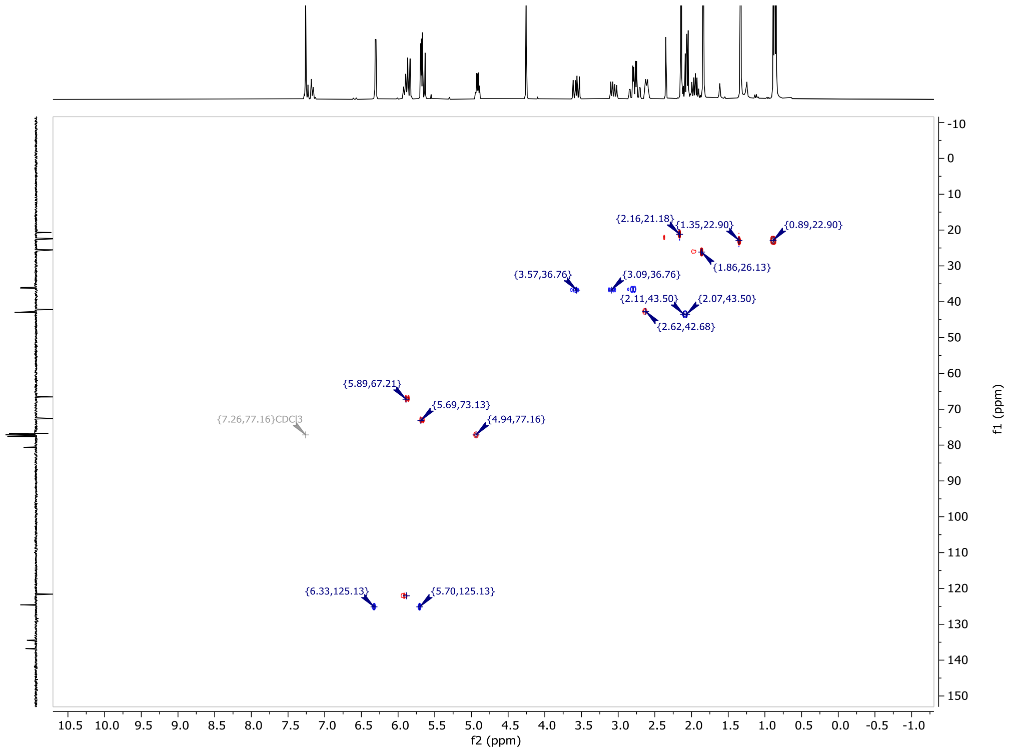

Figure S10. HSQC spectra of Lobatin A (**C2**).

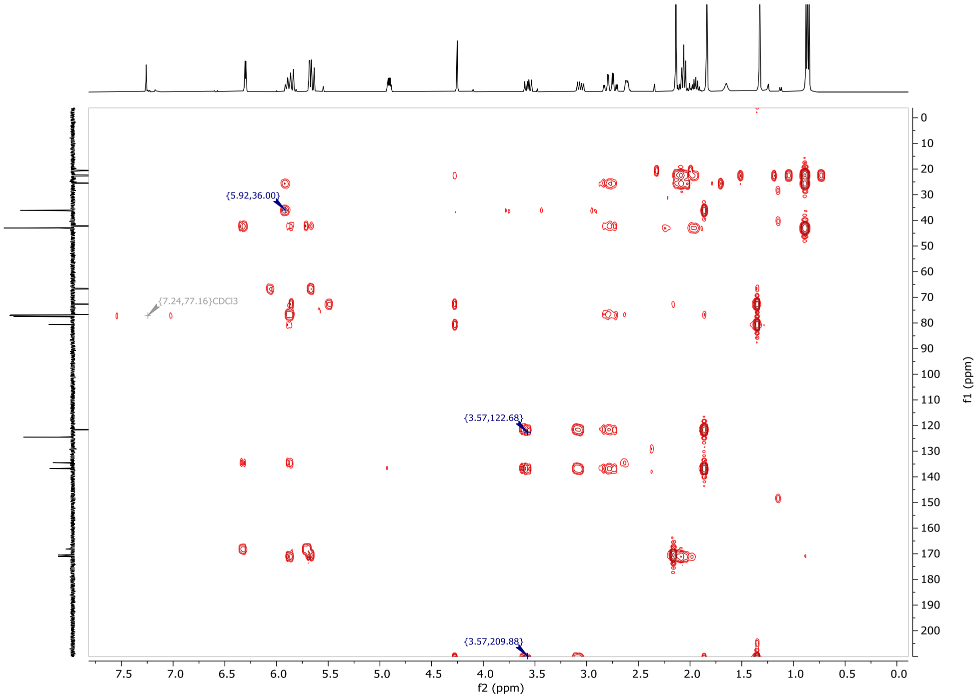

Figure S11. HMBC spectra of Lobatin A (**C2**).

molecules

Table S2. ^{1}H and ^{13}C NMR spectroscopic data of Lobatin A (**C2**) and comparison with literature values.

Signal	Observed δ		Literature δ [22]	
	^{1}H NMR(J-Hz)	^{13}C NMR	^{1}H NMR (J-Hz)	^{13}C NMR
**1**		210.1		210.6
**2**	3.57-3.06 dd (7.9-9.6-15.9)	36.0	3.57-3.07 dd (8.1-9.6-15.9)	36.0
**3**	5.91 t	121.5	5.90 t	121.4
**4**		136.8		136.8
**5**	2.82-2.73 dd (3.6-4.2-15.1)	42.9	2.83-2.74 dd (3.6-4.2-15.1)	42.9
**6**	4.92 td (3.8-7.7)	72.6	4.92 td	72.5
**7**	2.61 m (1.6-3.1-7.8)	42.2	2.62 m	42.1
**8**	5.85 dd (10.4)	76.6	5.86 dd (10.5)	76.6
**9**	5.65 d (10.4)	76.7	5.66 d (10.5)	76.6
**10**		80.6		80.5
**11**		134.4		134.3
**12**		168.2		168.3
**13**	6.31-5.69 d (2.9-3.4)	124.4	6.31-5.69 d (2.88-3.27)	124.4
**14**	1.33 s	25.6	1.33 s	25.5
**15**	1.84 s	22.6	1.85 s	22.5
**1´**		170.5		170.5
**2´**	2.12-2.08 m	42.1	2.11-2.06 m	42.1
**3´**	1.95 m	25.5	1.96 m (6.74)	25.4
**4´**	0.88 d (4.4)	22.5	0.88 d (6.7)	22.3
**5´**	0.85 d (4.5)	22.4	0.86 d (6.7)	22.3
**1´´**		171.0		170.8
**2´´**	2.14 s	20.7	2.15 s	20.5

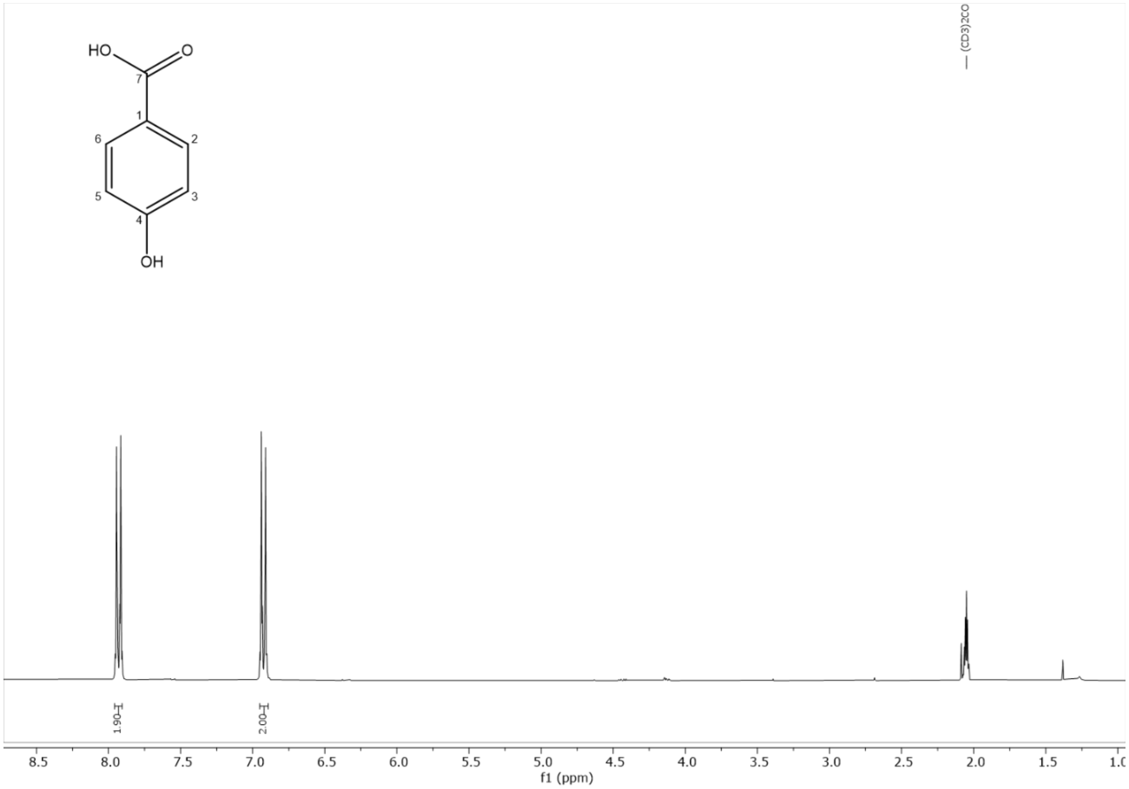

Figure S12. ^{1}H-NMR spectrum of p-Hydroxybenzoic acid (**C3**), (400 MHz, acetone-d_6).

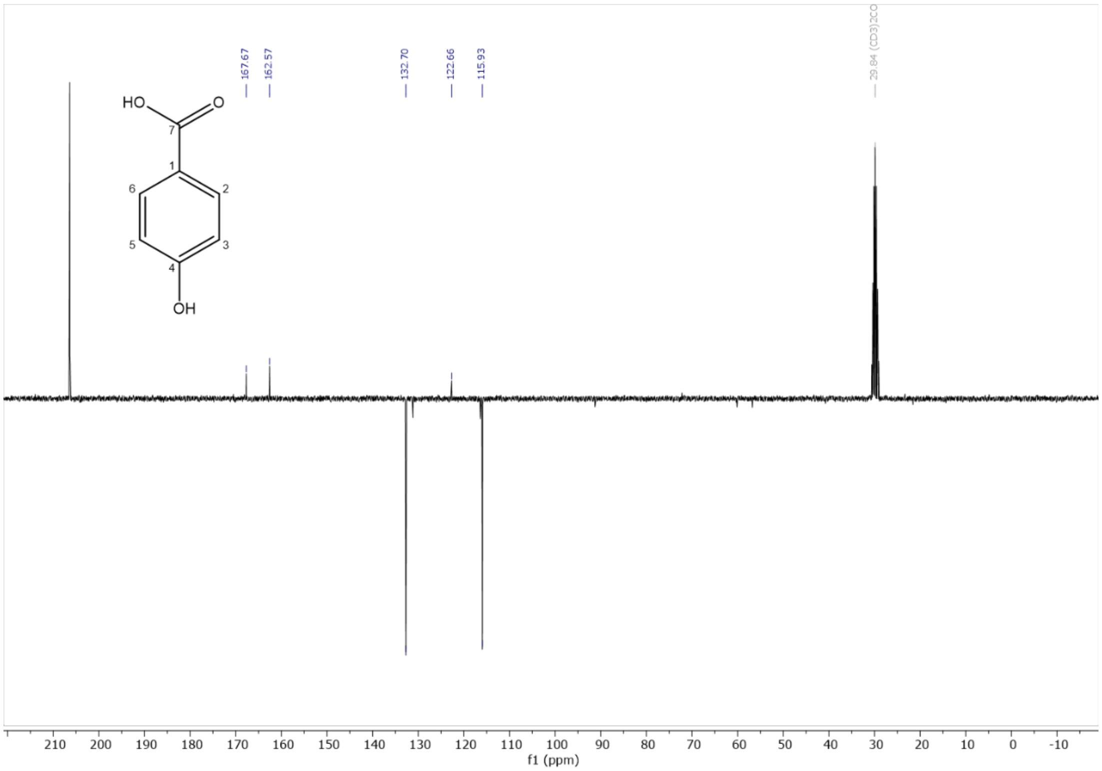

Figure S13. APT spectrum of p-Hydroxybenzoic acid (**C3**), (100 MHz, acetone-d_6).

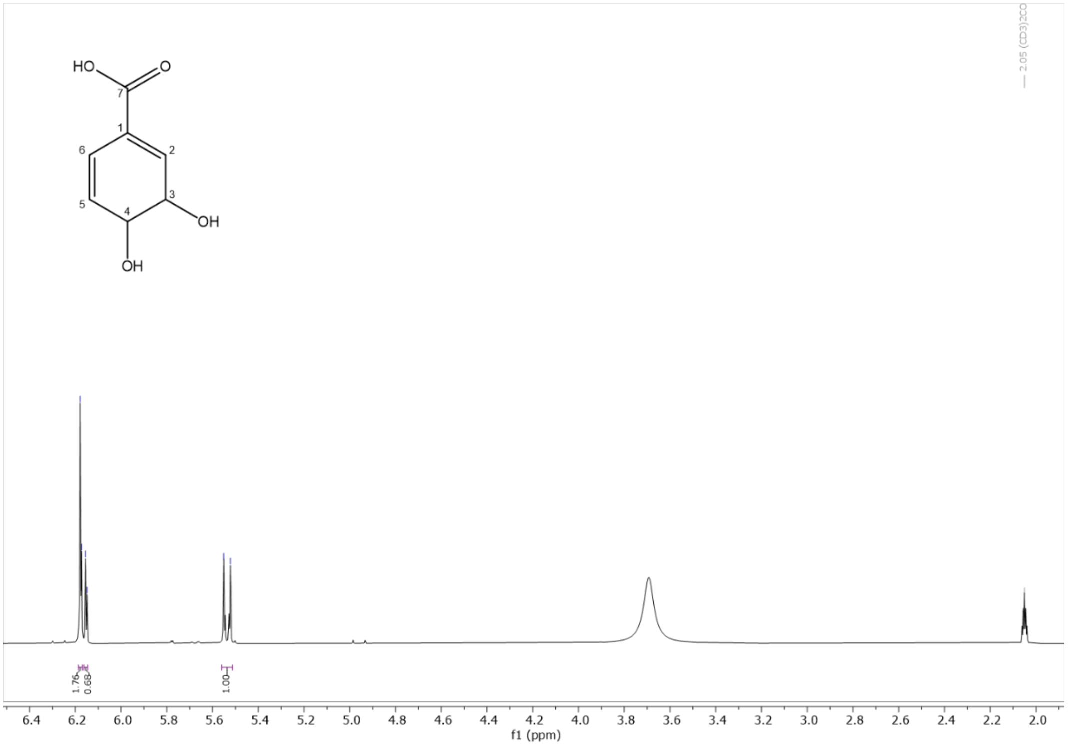

Figure S14. ^{1}H-NMR spectrum of 3,4-Dihydroxybenzoic acid (**C4**), (400 MHz, CD_3OD).

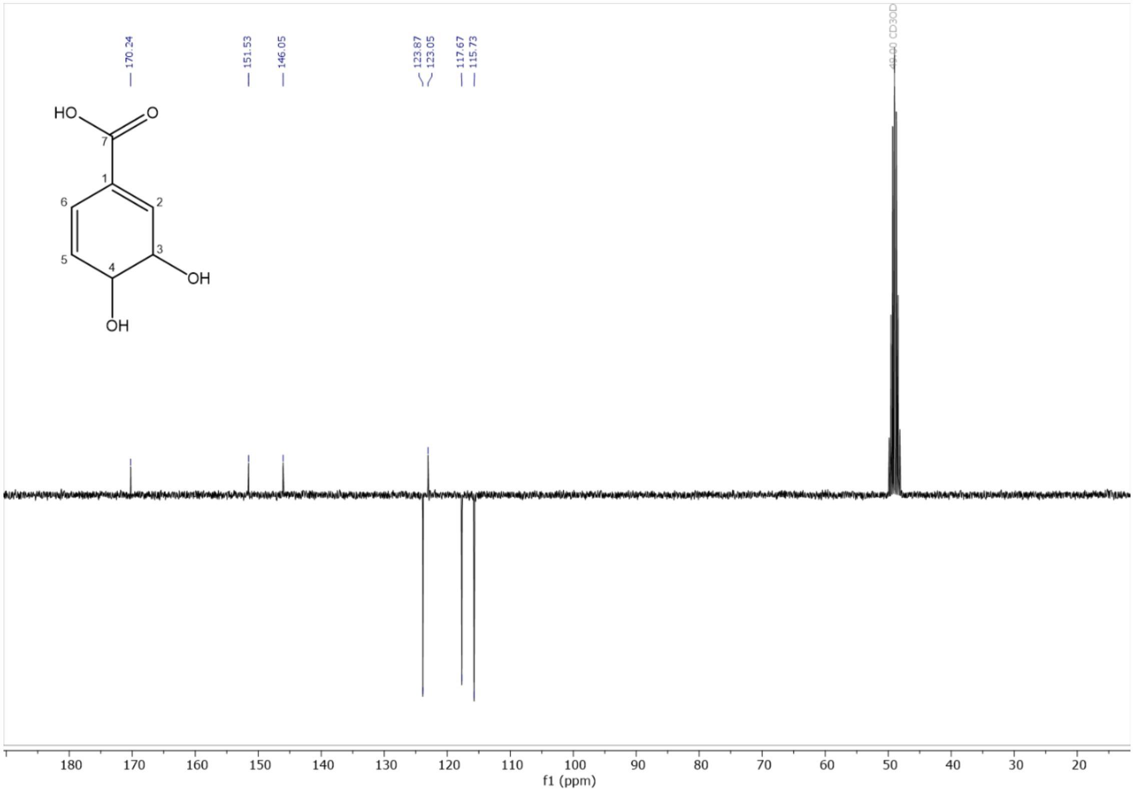

Figure S15. APT spectrum of 3,4-Dihydroxybenzoic acid (**C4**), (100 MHz, CD_3OD).

molecules

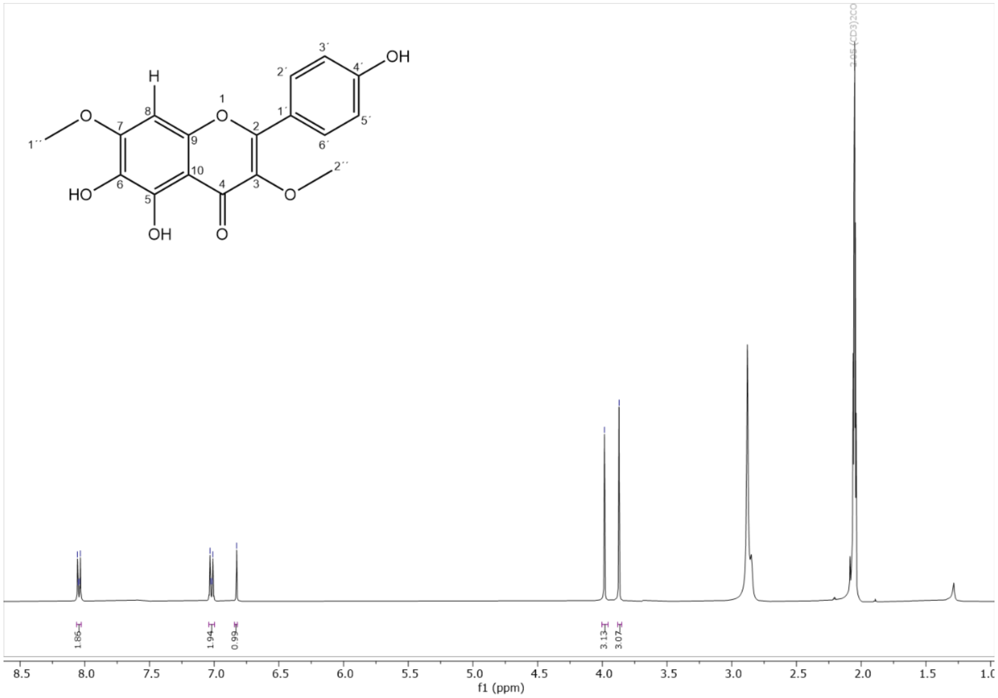

Figure S16. ^{1}H-NMR spectrum of 6-Hydroxykaempferol 3,7-dimethyl ether (**C5**), (400 MHz, acetone-d_6).

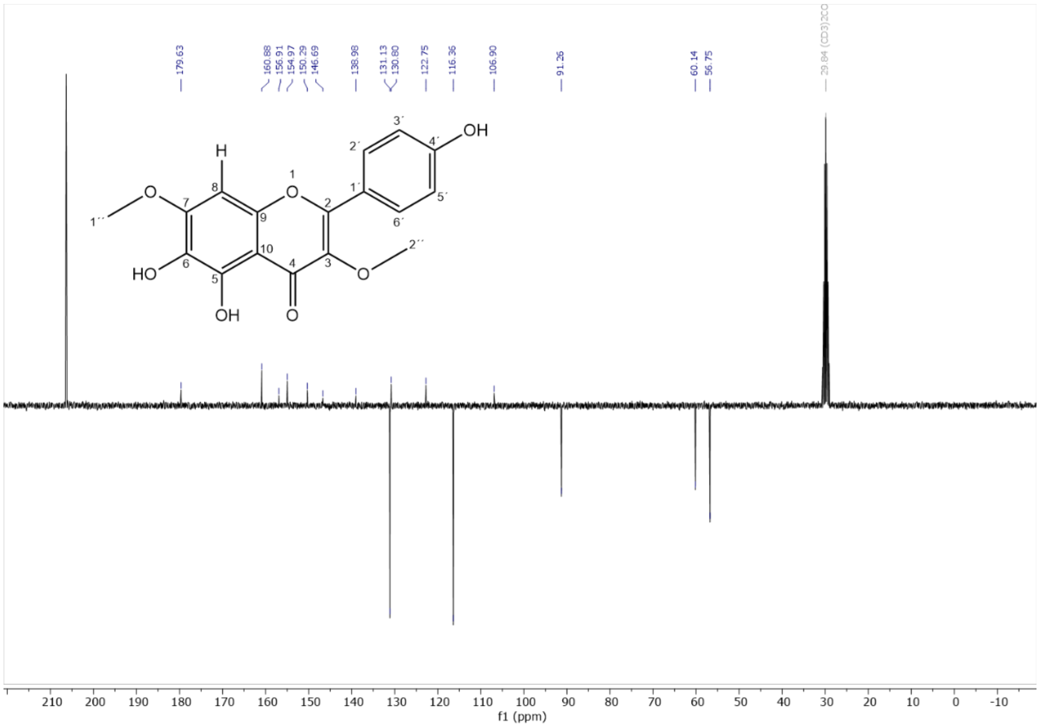

Figure S17. APT spectrum of 6-Hydroxykaempferol 3,7-dimethyl ether (**C5**), (100 MHz, acetone-d_6).

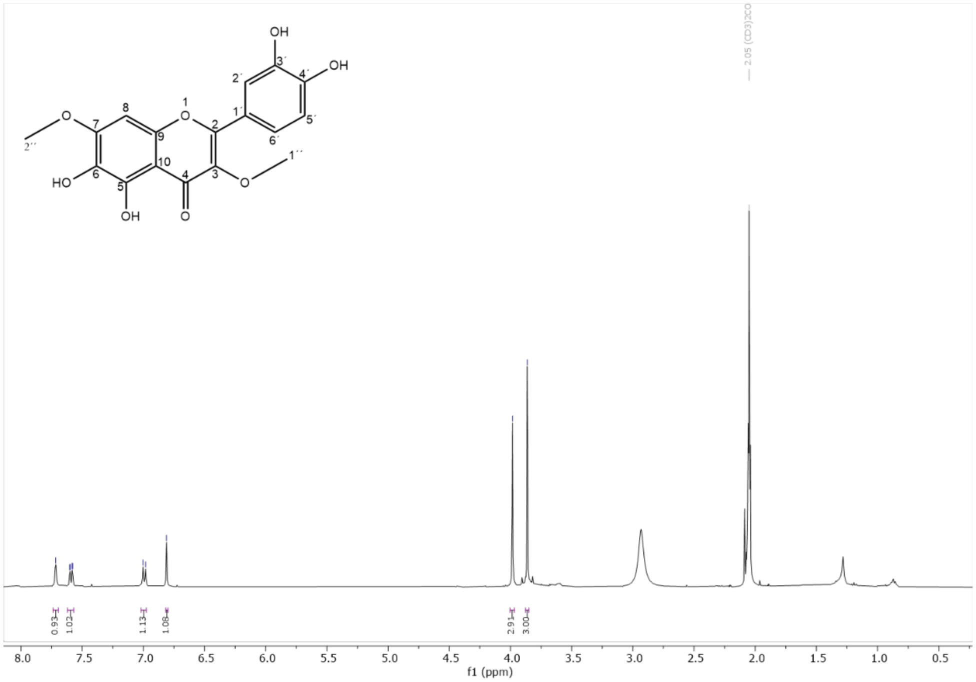

Figure S18. ^{1}H-NMR spectrum of Quercetagetin 3,7-dimethyl ether (**C6**), (400 MHz, acetone-d_6).

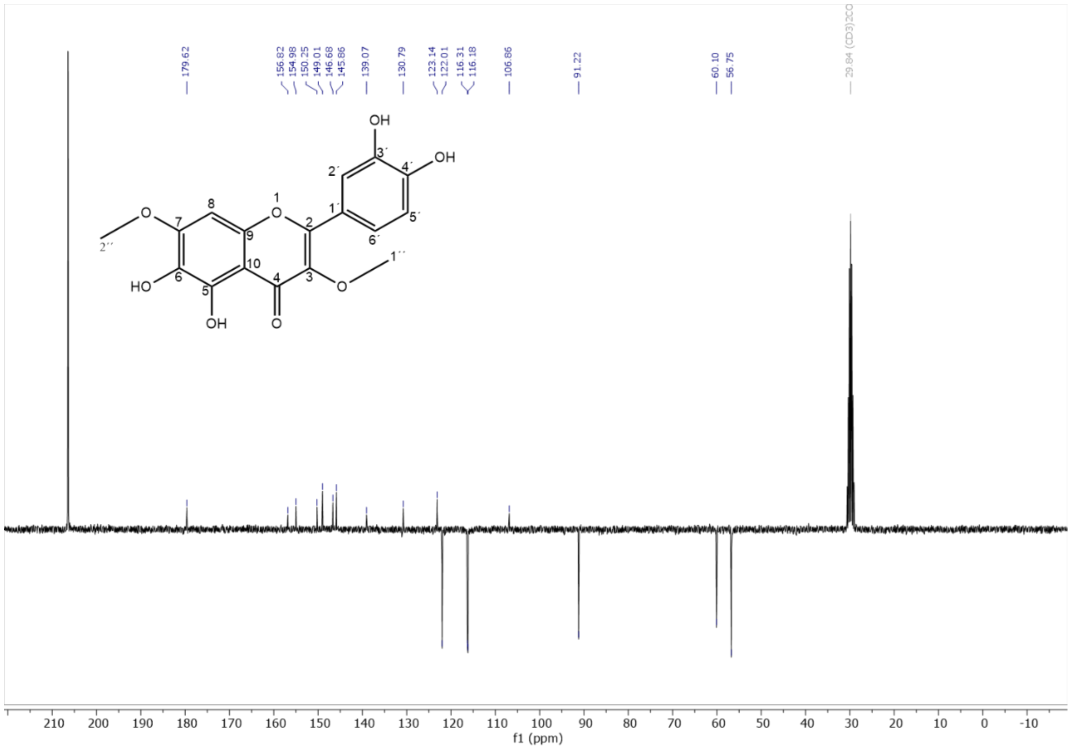

Figure S19. APT spectrum of Quercetagetin 3,7-dimethyl ether (**C6**), (100 MHz, acetone-d_6).

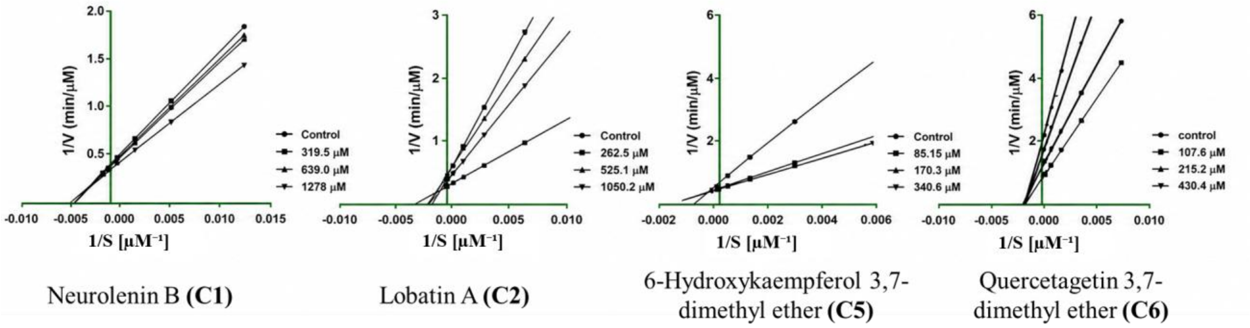

Figure S20. Lineweaver–Burk plots for apparent inhibition profiles on α-glucosidase (AG) by **C1**, **C2**, **C5** and **C6.**

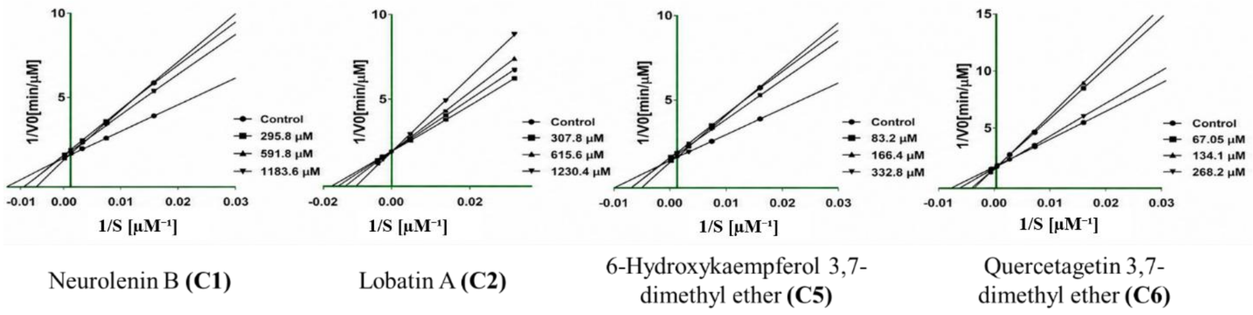

Figure S21. Lineweaver–Burk plots for apparent inhibition profiles on pancreatic lipase (PL) by **C1**, **C2**, **C5** and **C6.**

molecules

Table S3. Apparent Michaelis–Menten parameters and Ki estimates for the inhibition of pancreatic lipase (PL) by **C1**, **C2**, **C5** and **C6**.

Compound	Inhibitor level	Inhibitor concentration (µM)	Apparent Km (µM), 95% CI	Apparent Vmax, 95% CI	Estimated Ki (µM)	Graphically inferred inhibition profile
E	0 × IC_{50}	0	115.2 (104.5 - 127.0)	0.7839 (0.76 - 0.80)	—	—
C1	0.5 × IC_{50}	295.9	232.1 (204.5 - 263.7)	0.7785 (0.74 - 0.81)	285.5 ± 1.3	Competitive
C1	1.0 × IC_{50}	591.8	350.3 (307.1 - 400.5)	0.7784 (0.73 - 0.82)		Competitive
C1	2.0 × IC_{50}	1183.6	586.9 (507.6 - 682.7)	0.7787 (0.72 - 0.84)		Competitive
C2	0.5 × IC_{50}	307.8	228.9 (201.7 - 260.0)	0.7785 (0.74 - 0.81)	305.5 ± 1.8	Competitive
C2	1.0 × IC_{50}	615.6	343.8 (301.4 - 393.0)	0.7786 (0.73 - 0.82)		Competitive
C2	2.0 × IC_{50}	1231.2	573.5 (496.5 - 666.5)	0.7786 (0.72 - 0.84)		Competitive
C5	0.5 × IC_{50}	83.2	612.8 (540.2 - 698.0)	0.7785 (0.74 - 0.81)	83.2 ± 2.2	Competitive
C5	1.0 × IC_{50}	166.4	912.5 (793.5 - 1057)	0.7786 (0.73 - 0.82)		Competitive
C5	2.0 × IC_{50}	332.8	1538 (1288 - 1869)	0.7788 (0.72 - 0.84)		Competitive
C6	0.5 × IC_{50}	67.0	609.4 (537.3 - 694.1)	0.7785 (0.74 - 0.82)	67.0 ± 1.0	Competitive
C6	1.0 × IC_{50}	134.1	917.8 (798.0 - 1064)	0.7785 (0.77 - 0.83)		Competitive
C6	2.0 × IC_{50}	268.2	1538 (1288 - 1869)	0.7788 (0.73 - 0.84)		Competitive

Apparent Km and Vmax values (95% CI) were obtained from individual Michaelis–Menten fits. Apparent Ki values were estimated by global nonlinear regression in GraphPad Prism 8 using the complete substrate–inhibitor dataset and the model corresponding to the assigned apparent profile. All concentrations are final well concentrations, and Ki values should be interpreted as model-dependent estimates under the assay conditions. Apparent Vmax values are expressed as blank-corrected absorbance units at 405 nm (A405) obtained from endpoint assays.

molecules

Table S4. Apparent Michaelis–Menten parameters and Ki estimates for the inhibition of α-glucosidase (AG) by **C1**, **C2**, **C5** and **C6**.

Compound	Inhibitor level	Inhibitor concentration (μM)	Apparent Km (μM), 95% CI	Apparent Vmax, 95% CI	Estimated Ki (μM)	Graphically inferred inhibition profile
E	0 × IC_{50}	0	303.8 (197.4 - 474.3)	0.7557 (0.65 - 0.90)	—	—
C1	0.5 × IC_{50}	319.5	609.4 (537.3-694.1)	0.7555 (0.71 - 0.81)		Competitive
C1	1.0 × IC_{50}	639.0	917.8 (798.0 - 1064)	0.7570 (0.70 - 0.8)	318.2 ± 0.9	Competitive
C1	2.0 × IC_{50}	1278	1538 (1288 - 1869)	0.7597 (0.68 - 0.86)		Competitive
C2	0.5 × IC_{50}	262.5	640.8 (563.6 - 731.8)	0.7575 (0.71 -0.81)		Competitive
C2	1.0 × IC_{50}	525.1	972.5 (841.7 -1133)	0.7549 (0.67 - 0.82)	238.7 ± 1.28	Competitive
C2	2.0 × IC_{50}	1050.2	1640 (1370 - 2002)	0.7550 (0.68 to 0.86)		Competitive
C5	0.5 × IC_{50}	85.15	612.8 (540.2 - 698.0)	0.7582 (0.71 - 0.80)		Competitive
C5	1.0 × IC_{50}	170.3	912.5 (793.5 – 1057)	0.7549 (0.70 - 0.82)	85.0 ± 2.8	Competitive
C5	2.0 × IC_{50}	340.6	1538 (1288 – 1869)	0.7597 (0.67 - 0.86)		Competitive
C6	0.5 × IC_{50}	107.6	308.8 (277.0 - 389.2)	0.7185 (0.70 - 0.80)		Noncompetitive
C6	1.0 × IC_{50}	215.2	301.6 (267.0 - 345.2)	0.6705 (0.63 - 0.73)	107.2 ± 2.9	Noncompetitive
C6	2.0 × IC_{50}	430.4	312.1 (242.0 - 359.1)	0.4988 (0.41 - 0.55)		Noncompetitive

Apparent Km and Vmax values (95% CI) were obtained from individual Michaelis–Menten fits. Apparent Ki values were estimated by global nonlinear regression in GraphPad Prism 8 using the complete substrate–inhibitor dataset and the model corresponding to the assigned apparent profile. All concentrations are final well concentrations, and Ki values should be interpreted as model-dependent estimates under the assay conditions. Apparent Vmax values are expressed as blank-corrected absorbance units at 405 nm (A405) obtained from endpoint assays.
